# Mucin alleviates colonic barrier dysfunction by promoting spermine accumulation through enhanced arginine metabolism in *Limosilactobacillus mucosae*

**DOI:** 10.1128/msystems.00246-24

**Published:** 2024-04-02

**Authors:** Xingjian Zhou, Qian Xu, Xiangyu Zhang, Hao Wang, Yu Bai, Yujun Wu, Xiaoyi Liu, Zhenyu Wang, Jie Hu, Mingyi Huang, Yu Pi, Jinbiao Zhao, Junjun Wang, Dandan Han

**Affiliations:** 1 State Key Laboratory of Animal Nutrition and Feeding, College of Animal Science and Technology, China Agricultural University, Beijing, China; 2 Key Laboratory of Feed Biotechnology of the Ministry of Agriculture and Rural Affairs, Institute of Feed Research, Chinese Academy of Agricultural Sciences, Beijing, China; University of California San Diego, La Jolla, California, USA

**Keywords:** mucin, gut barrier function, *Limosilactobacillus mucosae*, spermine, arginine metabolism

## Abstract

Dietary fiber deprivation is linked to probiotic extinction, mucus barrier dysbiosis, and the overgrowth of mucin-degrading bacteria. However, whether and how mucin could rescue fiber deprivation-induced intestinal barrier defects remains largely unexplored. Here, we sought to investigate the potential role and mechanism by which exogenous mucin maintains the gut barrier function. The results showed that dietary mucin alleviated fiber deprivation-induced disruption of colonic barrier integrity and reduced spermine production *in vivo*. Importantly, we highlighted that microbial-derived spermine production, but not host-produced spermine, increased significantly after mucin supplementation, with a positive association with upgraded colonic *Lactobacillus* abundance. After employing an *in vitro* model, the microbial-derived spermine was consistently dominated by both mucin and *Lactobacillus* spp. Furthermore, *Limosilactobacillus mucosae* was identified as an essential spermine-producing *Lactobacillus* spp., and this isolated strain was responsible for spermine accumulation, especially after adhering to mucin *in vitro*. Specifically, the mucin-supplemented bacterial supernatant of *Limosilactobacillus mucosae* was verified to promote intestinal barrier functions through the increased spermine production with a dependence on enhanced arginine metabolism. Overall, these findings collectively provide evidence that mucin-modulated microbial arginine metabolism bridged the interplay between microbes and gut barrier function, illustrating possible implications for host gut health.

**IMPORTANCE:**

Microbial metabolites like short-chain fatty acids produced by dietary fiber fermentation have been demonstrated to have beneficial effects on intestinal health. However, it is essential to acknowledge that certain amino acids entering the colon can be metabolized by microorganisms to produce polyamines. The polyamines can promote the renewal of intestinal epithelial cell and maintain host-microbe homeostasis. Our study highlighted the specific enrichment by mucin on promoting the arginine metabolism in *Limosilactobacillus mucosae* to produce spermine, suggesting that microbial-derived polyamines support a significant enhancement on the goblet cell proliferation and barrier function.

## INTRODUCTION

Emerging evidence has revealed long-term western diet had a negative effect on intestinal homeostasis while dietary fiber is a crucial diet supplement for maintaining the normal intestinal mucosal function, which is indispensable for commensal colonization and pathogen-immune homeostasis (1, 2). However, as the western diet is under low dietary fiber conditions, the abundance of mucin-degrading bacteria, including mucin-degrading specialists and generalists, has rapidly increased. Specifically, dietary fiber deprivation induces the breakage of the intestinal epithelial barrier, which is linked with the overgrowth of bacteria capable of degrading mucin (3–5). The intestinal mucus along the gastrointestinal tract provides unique ecological niches for various bacteria (6). The mucus layer is mainly composed of mucin, which is produced and secreted by intestinal goblet cells, and primarily consists of heavily glycosylated mucin 2 protein-rich O-glycosylation (7–9). Mucin, a microbial accessible glycoprotein *in vivo*, has been identified to suppress pathogen colonization (10), by influencing the physiological behavior of microbes, including surface adhesion and quorum sensing, as well as facilitating gut microbes to maintain immune homeostasis (11, 12). Therefore, mucin has been shown to improve gut function by altering the gut microbiota community and regulating complex host-microbiome interactions (13).

The metabolites derived from gut microbiome play an essential role in regulating the host intestinal health. Polyamines, such as putrescine, spermidine, and spermine, are low-molecular weight, organic polycations that are synthesized from amino acid precursors including arginine and ornithine in host cells or gut microbes (14–16). Recently, extensive research has focused on the potential beneficial effects of polyamines on the regulation of host physiology which included promoting intestinal epithelial cell proliferation (17). The increasing concentration of polyamines stimulates the gut mucosal renewal and enhances barrier function (18, 19). The intestinal microbiota-derived spermine could shape the host-microbiome interface (20, 21). Moreover, dietary spermine supplementation can also enhance intestinal development, improve immune functions, and upgrade the abundance of probiotics in the colon of weaned piglets (22). Alterations in the composition of intestinal microorganisms including *Bacteroides*, *Escherichia*, *Prevotella*, and *Lactobacillus* were recently demonstrated to produce polyamines, while *Lactobacillus* was thought to be the predominant bacterium contributing to polyamines synthesis in mammals’ intestine (20, 23). Presently, little is known about the involvement of gut microbes in mucin-mediated alleviation of intestinal barrier defects. In particular, it is unclear whether intestinal microorganisms alter polyamine metabolic properties by interacting with mucin.

Therefore, the objective of this study was to investigate how mucin intervention critically influences gut health by shaping the gut microbiota and promoting probiotic colonization to rescue the dysbiosis of the intestinal barrier function through bacterial metabolites. To further explore the interactions between mucin and microbiota in a controlled manner. We used both mucin-fed mice combined with *in vitro* fermentation system, and a co-culture model of microbes and colonic goblet cells to investigate the involvement of key bacteria enriched by mucin in the beneficial effects on the intestinal barrier function.

## MATERIALS AND METHODS

### Mice and experimental design

The animal study was approved by the Care and Use of Experimental Animal Committee of China Agricultural University (AW20602202-1-3), and all protocols were conducted according to the standards of animal welfare of China Agricultural University. Seven-week-old C57BL/6 male mice were purchased from SPF Biotechnology Co. Ltd. (Beijing, China). All mice were raised at 22°C–24°C and 40%–50% relative humidity with a 12 h light/dark cycle and food and water *ad libitum*. After 1 week of acclimation to a standard diet, mice were randomly assigned to three diets: a fiber-containing diet (FC), a fiber-free diet (FF), and a fiber-free diet with mucin supplement (MUC). The ingredients of the three diets are listed in Table S1. The feeding trial lasted for 4 weeks. In the second animal trial, two mouse groups (FF*Lm* and MUC*Lm*) were supplemented with *Limosilactobacillus mucosae* daily by oral gavage in 0.2 mL phosphate-buffered saline (PBS) containing 10^9^ CFU/mL bacteria. After sacrificing by dislocation, colonic tissue and digesta were collected from the mice and stored at −80°C for further analysis.

### *In vitro* fermentation trial

The substrates and medium used were as follows: cellulose (Pioneer Biotech Company, Xi’an, China), Mucin Type II (Sigma-Aldrich, Missouri, USA), bacterial peptone (the amino acid composition was analyzed in duplicated in Fig. S1), bacterial amino acid broth, and arginine deficiency broth (Coolaber Science & Technology Co. Ltd., Beijing, China); galactose (Gal), fucose (Fuc), N-acetyl-galactosamine (GalNAc), N-acetylglucosamine (GlcNAc), and N-acetylneuraminic acid (Neu5Ac) (Macklin Inc., Shanghai, China); and sardomozide dihydrochloride (MedChemExpress, New Jersey, USA).

The inoculum was prepared according to our previously published study (24). Briefly, 10 healthy pigs (Duroc × Landrace × Yorkshire, 5 boars and 5 gilts, body weight: 35 kg approximately) were selected from the FengNing Swine Research Unit of China Agricultural University (Chengdejiuyun Agricultural and Livestock Co. Ltd., Hebei, China). The pigs were fed a standard corn-soybean meal, and no antibiotics were used 3 months before fecal collection. The donors’ feces were snap frozen after immediate mixing with sterile 15% glycerol-phosphate buffers at a ratio of 1:10 (m/vol) and then stored at −80°C.

The *in vitro* batch fermentation was conducted according to a previous protocol with a few modifications (24, 25). The medium containing 15 g/L bacterial peptone was dissolved in PBS buffer and 50 mL of reductive solution/L of peptone buffer (312 mg of cysteine and 312 mg of sodium sulfide in 2 mL of 1 M NaOH). The buffer was then autoclaved at 121°C for 15 min. The inoculated fecal source was thawed at 39°C and centrifuged at 2,000 rpm for 3 min to remove solid particles from the feces. The inoculation was performed in an anaerobic handling box. The *in vitro* fermentation trial lasted 24 h on a thermostatic shaker (39°C, 200 rpm). After incubation, the fermentation broth supernatant was collected and stored at −80°C.

### Bacterial and cell culture

*Limosilactobacillus mucosae* was isolated and kept in our laboratory from the feces of pigs fed with a fiber-rich diet from the same research unit. *L. mucosae* was cultured in MRS (deMan, Rogosa, and Sharpe broth) medium (Con) or additionally with 1%mucin (Mucin) or with all glucose replaced by mucin (Mucin_Rep) or supplemented with 5% (m/vol) arginine, 1 µM ornithine, putrescine, or spermidine, respectively.

The cell model of MUC2 overexpression and inhibitory expression was established in human colonic cell line LS174T cells, they were divided into the control group (Con), the agonist group (PMA, phorbol 12-myristate 13-acetate, a mucin stimulator, 0.5 µM PMA used in this study), and the inhibitory group (BRNAC, bromelain and N-acetyl-L-cysteine can remove mucin from the cell surface, 25 µg/mL BR + 5 mM NAC used in this study) (26, 27). LS174T cells were seeded in 12-well plates at a cell density of 6 × 10^5^ cells/mL for 24 h. After treatment, the cells and their culture supernatants were collected and total RNA was extracted. In the co-culture experiment, LS174T cells were seeded in 6-well plates at a density of 3 × 10^6^ cells/mL and co-cultured with 1 × 10^7^ CFU/mL *L*. *mucosae* on cell slides in the same treatment, and MUC2 and 16S rRNA were fluorescently stained. For the bacterial supernatant treatment, the supernatant was filtered through a 0.22-µm filter membrane (Millipore, Massachusetts, USA) after pH was adjusted to 7.0, and 10% (vol/vol) of the supernatant was added to each well for cell treatment.

### Quantitative analysis of metabolites

A mixed standard solution of polyamines was prepared using tryptamine, cadaverine, histamine, tyramine putrescine, spermidine, and spermine (Solarbio Science & Technology Co. Ltd., Beijing, China). The polyamine determination was based on a previous study with slight modifications (28). The derivatization reaction was carried out by adding 2 mL of dansyl chloride solution to 2 mL of supernatant and adjusted to pH 9–10 with 200 µL of 2 M NaOH and 300 µL of Na_2_CO_3_-saturated solution. The mixture was then heated in a dark water bath for 45 min at 45°C. After the reaction, 100 µL of ammonia was added to stop the derivatization. Acetone was removed under a slight stream of N_2_. Before high-performance liquid chromatography (HPLC) analysis, a filtration through a 0.22-µm membrane filter (Millipore, Massachusetts, USA) was performed. HPLC determination of the polyamines was performed with an Agilent 1260 Infinity II LC System coupled with a DAD detector and a column (RP-C18, 250 × 4.6 mm, 5 µm, Waters, Massachusetts, USA). Each injection consisted of 10 µL of the sample solution. A wavelength of 254 nm was used for this study. The mobile phase comprised ammonium acetate (A) and acetonitrile (B). The HPLC gradient elution for the polyamine analysis is shown in Table S2.

The bacterial culture was centrifuged at 5,000 rpm for 10 min to obtain the supernatant. Metabolome analysis of bacterial supernatants was performed using a UHPLC-Q Exactive HF-X system (Thermo Fisher Scientific, Massachusetts, USA), and samples were separated using an HSS T3 column (100 mm × 2.1 mm i.d., 1.8 µm, Waters, Massachusetts, USA) and then analyzed by mass spectrometry. Data were analyzed using the online platform of Majorbio cloud platform (cloud.majorbio.com). The selection of significantly different metabolites was determined based on the variable importance in the projection (VIP) obtained by the OPLS-DA model and the *P* value of Student’s *t*-test; the metabolites with VIP > 1 and *P* < 0.05 were significantly different metabolites. For the KEGG pathway analysis, the relative-betweenness centrality algorithm was used to obtain the pathway that was significantly enriched in the metabolite set, and the false discovery rate (FDR) withBenjamini-Hochberg (BH) procedure was used to correct the *P* value. When the corrected *P* < 0.05, it was considered that this pathway was significantly enriched.

### RT-qPCR

Total RNA was extracted using the RNApure Bacteria Kit (CWbiotech Co. Ltd., Jiangsu, China) according to the manufacturer’s protocol. The extracted RNA was quantified using NanoDrop 2000 (Thermo Fisher Scientific, Massachusetts, USA) and then diluted to the same concentration. cDNA was obtained from bacterial RNA using a QuantiTect Reverse Transcription Kit (Tsingke Biotechnology Co. Ltd., Beijing, China). Quantitative PCR (qPCR) was performed using a Riche LightCycler 96 Real-Time PCR System (Roche, Basel, Switzerland). The gene primers for mice (*MUC2*, *Ogt*, *Fut2*, *C1gal*, *ST6galnac2*, *ODC*, *SAM*, *SPD*, and *SPM*) and arginine metabolism genes (*arcA*, *arcB*, *arcC*, and *arcD*) for *L. mucosae* are listed in Table S3. We calculated the relative expression of target genes relative to that of a housekeeping gene (*β-actin* in mice and 16S rRNA in *Lactobacillus*) using the 2^−ΔΔCt^ method.

### Immunofluorescence and FISH staining

The mice colon tissue and LS174T cell bacteria co-culture smears were fixed in 4% paraformaldehyde at 4°C for 20 min, followed by a triple washing step with PBS. The presence of Mucin 2 glycoprotein was investigated using immunocytochemistry. Briefly, fixed cultures were permeabilized with 1% Triton X-100 blocking buffer (30 min) and primary antibody anti-mucin 2 mouse mAb (Servicebio Technology Co. Ltd., Wuhan, China) at 4°C overnight. Cy3-conjugated Goat Anti-mouse antibody (Servicebio Technology Co. Ltd.) was added for 2 h at 4°C, followed by nuclear counterstaining with 4′,6-diamidino-2-phenylindole (DAPI) for 1 min at room temperature. *Limosilactobacillus mucosae* ribosomal RNA (rRNA) fluorescence *in situ* hybridization (FISH) was performed using the EUB338 16S rRNA gene probe labeled with the 5′-Cy5 fluorophore label, and the sequence used was 5′-GCTGCCTCCCGTAGGAGT-3′ (29). Samples were observed using a Zeiss Axio Imager 2 system (Carl Zeiss, Jena, Germany).

### 16S rRNA sequencing analysis and absolute quantification

Total genomic DNA was extracted from the samples using a QIAamp Fast DNA Stool Mini Kit (Qiagen, Tübingen, Germany). The V3–V4 region of the 16S rRNA gene was amplified using universal primers, pooled into equimolar amounts, and sequenced on the Illumina MiSeq platform to generate paired-end reads of 300 bp. Quantitative Insights into Microbial Ecology 2 software was employed to perform microbial community analysis (30). Quality control and denoising were performed simultaneously using DADA2 with default parameters to generate ASVs. Only ASVs with a minimum abundance of two reads that were detected in more than two samples were retained. To avoid the bias resulting from variable sequencing depth, all samples were rarefied to the minimal sequence depth (31,789 reads). ASVs were taxonomically classified using the SILVA 138 database. The representative sequences of differential ASVs analyzed by Linear discriminant analysis Effect Size (LEfSe) analysis were further classified against the NCBI 16S rRNA database using the BLAST software (https://blast.ncbi.nlm.nih.gov). The total bacteria number and differential enrichment of microbiota on the specie level (i.e., *L. mucosae* and *Escherichia coli*) in mice were further verified by real time quantitative PCR (RT-qPCR) as previously described using species-specific primers (31).

### Microscopic observation *L. mucosae* adhering on mucin

*L. mucosae* was cultured for 8 h, harvested by centrifugation (4,000 × *g* for 5 min at 4°C), and resuspended to 1 × 10^6^ colony-forming units/mL. The glass slides were coated with mucin overnight, dried, and fixed with methanol for 20 min. The glass slide coated with mucin was placed in a culture dish filled with 20 mL of bacterial solution after the adjusted concentration (1 × 10^6^ CFU/mL), incubated (37°C for 1 h), washed five times with shaking in sterile normal saline to wash away the non-adherent bacteria, followed by natural drying, and then fixed with methanol for 20 min after drying. Bacteria were observed and counted under a microscope after Gram staining using a kit (Solarbio Science & Technology Co. Ltd. Beijing, China).

### Western blot analysis

The total surface protein was first extracted from *L. mucosae*, the bacterial surface protein was extracted after 8 h of culture, the substrate was collected by centrifugation at 8,000 *g* for 10 min at 4°C, after two slow rinses with PBS buffer, 5 M LiCl was added at a ratio of 1:5 for mixing and then shaken at 150 rpm for 45 min at 37°C, and the supernatant was collected at 10,000 *g* at 4°C for 10 min and filtered through a 0.22-µm filter membrane (Millipore, USA) (32). The concentration of total protein in the supernatant was quantified using the Pierce BCA Protein Quantification Kit (Thermo Fisher Science, USA), and then, the protein content of the sample was diluted to the same level. The primary antibody for *Lactobacillus* GAPDH protein was a kind gift from Prof. Haifeng Wang at Zhejiang University (Hangzhou, China). The capillary-based auto western blotting system was used to analyze the protein expression (Protein Simple Wes, USA). All the process was operated according to the manufacturer’s instructions. The data processing was analyzed using the Compass software.

### Cell viability analysis

The CellTiter-Glo luminescent cell viability assay (Promega Co., Madison, WI, USA) was used to measure LS174T cell viability in the bacterial supernatant. The experimental procedure was based on high-sensitivity bioluminescence detection technology, and the number of living cells and cell vitality in the culture were measured using ATP quantification.

### Statistical analyses

Statistical analysis was performed using the IBM SPSS Statistics software (version 22.0; IBM Co., New York, USA). Student’s *t*-test was used to detect statistical significance between the two treatment groups. One-way analysis of variance(ANOVA) and *Tukey’s* test were performed for multiple comparisons. Data are presented as mean ± standard error of the mean. Statistical significance is indicated as *P ˂ 0.05, **P ˂ 0.01, or ***P ˂ 0.001. Graphs were generated using the GraphPad Prism 8.0 software (GraphPad Software, California, USA).

## RESULTS

### Dietary mucin supplementation mitigates fiber deprivation-induced colonic barrier defects and fecal spermine reduction

**Fig 1:**
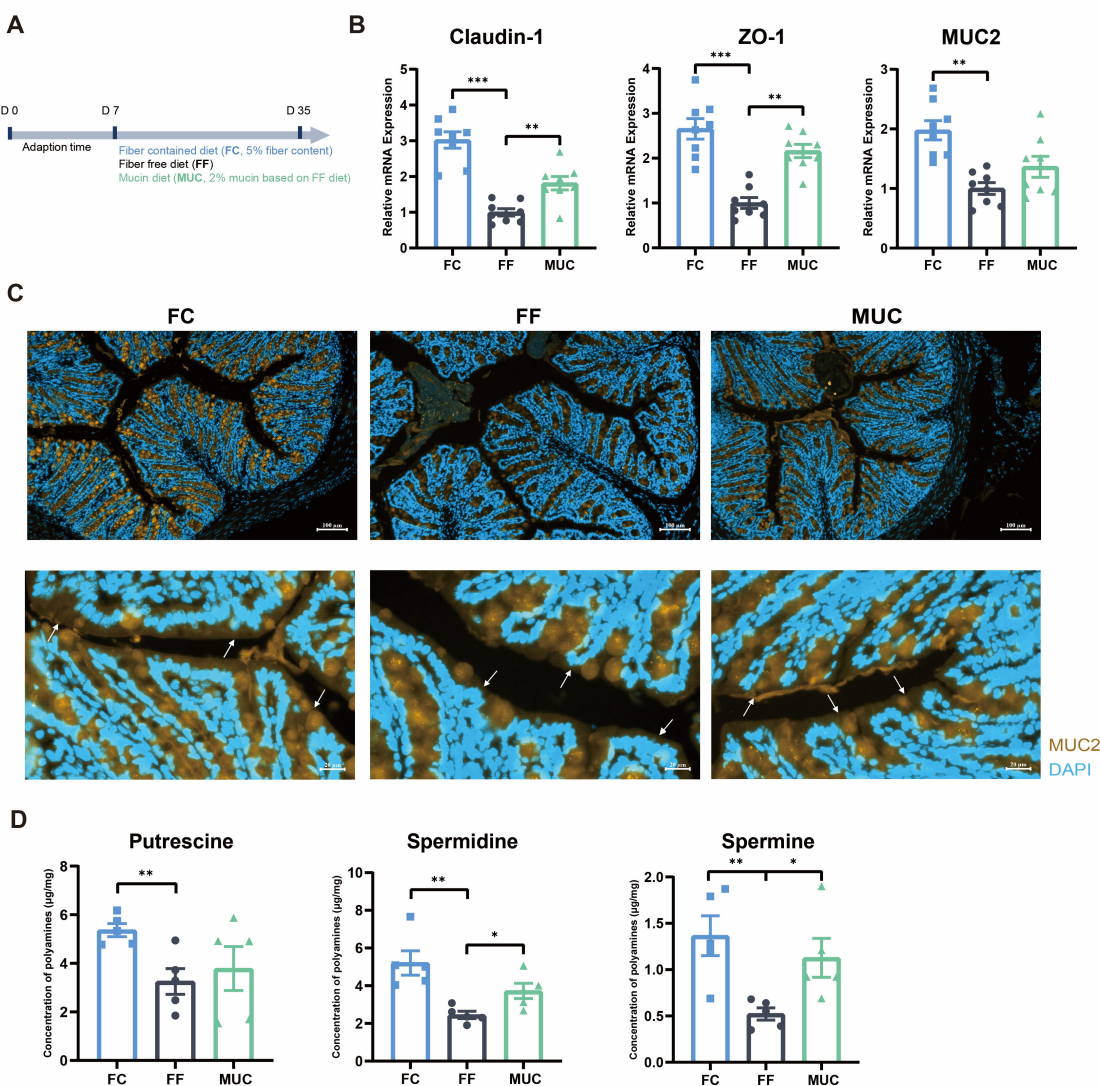
Mucin reverses the intestinal barrier dysfunction and spermine reduction in fiber deprivation mice. (**A**) Animal experiment set-up. (**B**) qPCR analysis of tight junction proteins *Claudin-1*, *Zo-1*, and *MUC2* from the colons of mice (*n* = 8). (**C**) Representative immunofluorescence staining for mucus of mice colons using *MUC2* (yellow) with DAPI (blue). (**D**) The concentrations of fecal putrescine, spermidine, and spermine and the sum in the mice (*n* = 5). Statistics was performed with one-way ANOVA, followed by Tukey’s multiple comparison test. *P < 0.05, **P < 0.01, and ***P < 0.001.

We and others have previously reported the disruption of the colonic mucus layer in response to fiber deprivation (33–35), and as such, we wished to determine whether mucin could reverse the intestinal defects in fiber-deprived mice. The mice were fed a FF or MUC (Fig. 1A). Compared with a standard diet rich in FC, fiber deprivation caused gut barrier dysfunction, as evidenced by the decreased mRNA expression of *Claudin1*, *ZO-1*, and *MUC2* (Fig. 1B), as well as reduced Mucin 2 secretion (Fig. 1C). Mucin supplementation based on the FF diet significantly relieved the damaged intestinal barrier function, as shown by the increased gene levels of *Claudin1*, *ZO-1* (Fig. 1B), and the higher Mucin 2 integrity layer (Fig. 1C). To further investigate the molecular basis of decreased mucin levels in dietary fiber-deprived mice (36), mucin-synthesizing enzymes in the colon were analyzed. The mRNA expression levels of *C1gal*, *Ogt*, and *ST6galnac2* were profoundly decreased in the colons of the FF group (Fig. S2). However, administration of mucin failed to rescue the gene levels of mucin-synthesizing enzymes, suggesting that the promoting effects of mucin supplementation on gut barrier functions were not achieved by upgrading host’s mucin synthesis. As the beneficial effects of polyamines on the gut epithelial renewal and intestinal barrier function had been reported by previous studies (17). The targeted profiling of fecal polyamines was performed using HPLC analysis. Among the eight common polyamines, only decreased levels of putrescine, spermidine, and spermine, produced from arginine metabolism, were observed in the FF group (*P* < 0.01; Fig. 1D), but not the concentrations of tryptamine, cadaverine, histamine, and tyramine (Fig. S3). Furthermore, mucin supplementation significantly enhanced the production of spermidine and spermine (*P* < 0.05; Fig. 1D), but not the putrescine level in the colon. In short, these results indicate that mucin is vital for protecting the host intestinal epithelial mucus barrier and spermine production induced by dietary fiber deprivation.

### Spermine accumulation is not achieved by host epithelium polyamine synthesis in mucin-fed mice

**Fig 2:**
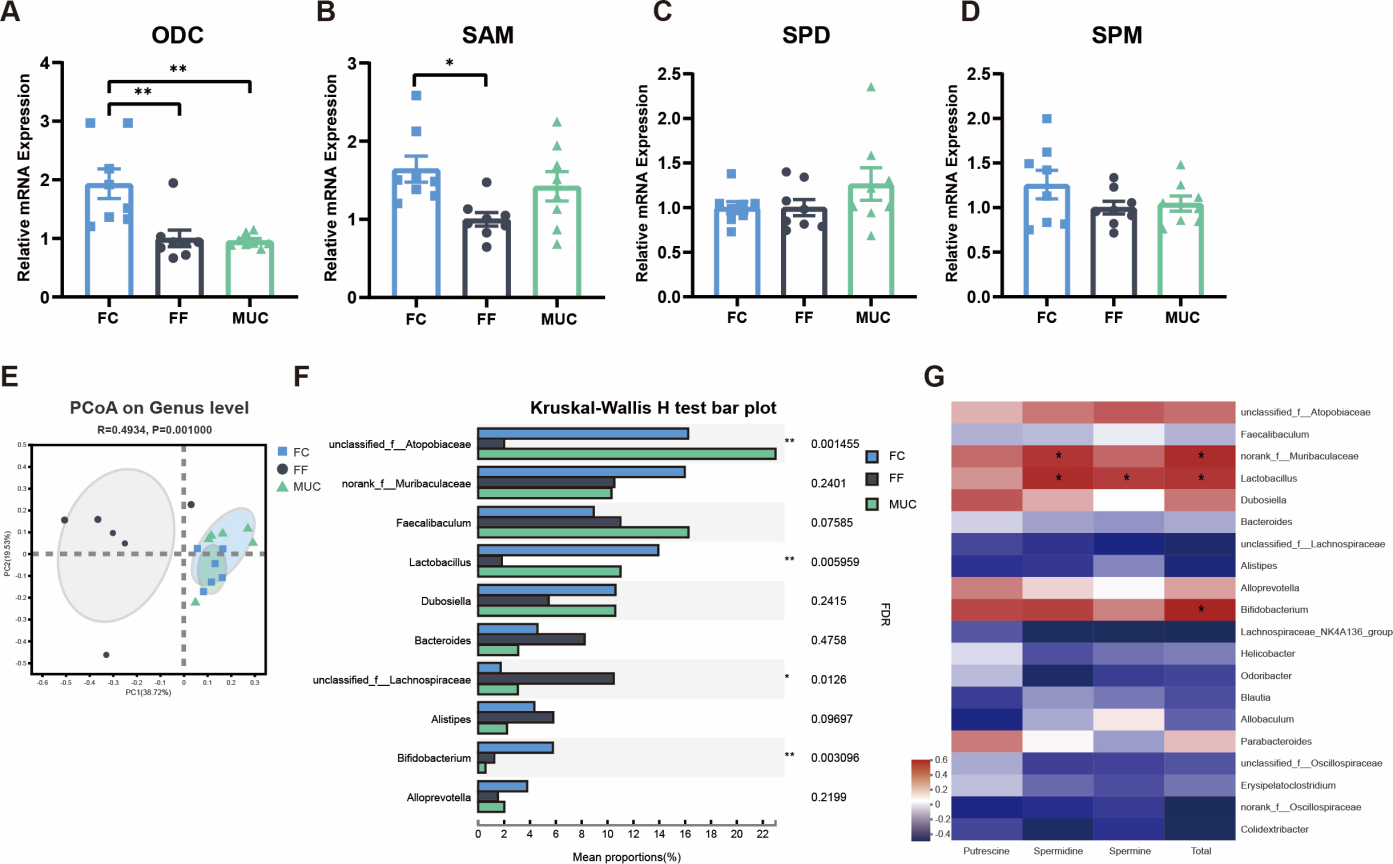
Mucin fails to elevate host-derived polyamine synthesis but recovers microbial communities in fiber-free mice. (**A–D**) qPCR analysis of *ODC*, *SAM*, *SPD*, and *SPM* from the colons of mice (*n* = 8) fed with FC, FF, and a fiber-free diet with MUC. Statistics was performed with one-way ANOVA, followed by Tukey’s multiple comparison test. (**E**) PCoA profile of the colon microbiome based on weighted the Bray-Curtis distance metrics at the genus level (Permutational multivariate analysis of variance (PERMANOVA), *P* = 0.001, *R*^2^ = 0.24). (**F**) Different bacterial abundance of the colon microbiome on genus level (*n* = 6). Statistical analyses were performed using Kruskal-Wallis test with *P* value adjustment using FDR correction. Significance between community structure was evaluated by PERMANOVA. (**G**) Heatmap between the polyamine concentrations and the relative abundance of top 20 genera in the colon. *P < 0.05, **P < 0.01, and ***P < 0.001.

To identify the source of elevated spermine, we first explored the effects of mucin supplementation on spermine synthesis-related enzymes in host intestinal epithelial cells. Ornithine decarboxylase (*ODC*) is the first rate-limiting enzyme in polyamine biosynthesis, and its expression and bioactivity directly affect the production of polyamines in cells (37). Fiber-free diet significantly downregulated colonic *ODC* mRNA expression compared with FC mice (*P* < 0.01), while mucin supplementation to FF did not alter the *ODC* gene level (Fig. 2A). Similar results were observed in the expression of S-adenosylmethionine synthetase (*SAM*) between FF diet-fed mice and MUC diet-fed mice (Fig. 2B). However, there was no difference in the expression of spermidine synthase (*SPD*) and spermine synthase (*SPM*), which directly influenced the concentration of polyamines in the cells (Fig. 2C and D). Therefore, we speculated that spermine produced by intestinal epithelial cells was not the main factor causing the difference in fecal spermine content and exogenous mucin addition failed to influence spermine synthesis in the host intestinal cells.

### Spermine synthesis is positively correlated with the enrichment of *Lactobacillus* in mucin-fed mice

**Fig 3:**
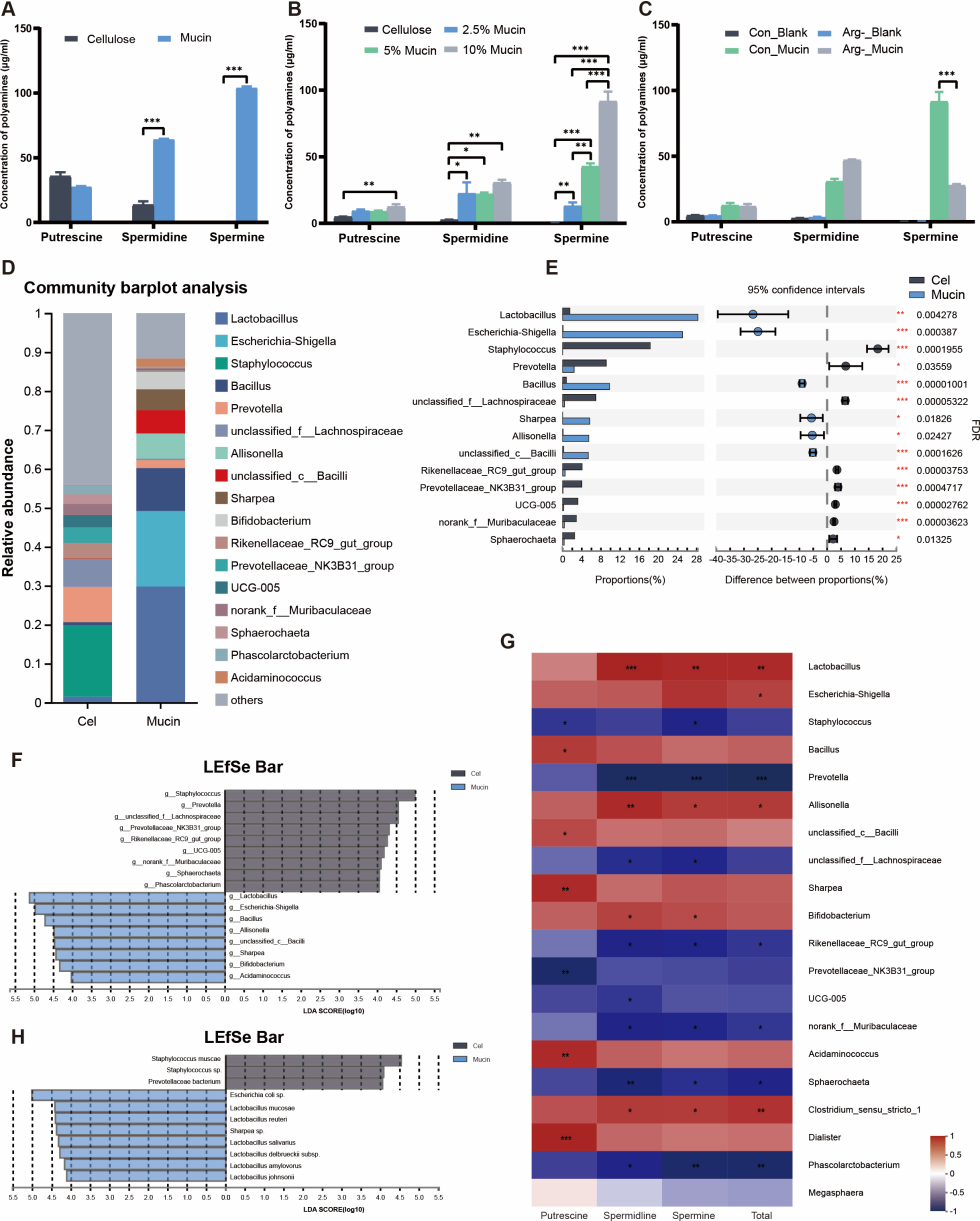
Mucin exclusively enhances the bacterial spermine production and enriches *Lactobacillus* species in *in vitro* fermentation. (**A**) Cellulose and Mucin modulated bacterial production of polyamines by *in vitro* fermentation. (**B**) Bacterial elevated production of polyamines by mucin followed the dose effect. (**C**) The full amino acid (Con) and arginine deprivation (Arg-) substrates in *in vitro* fermentation modulated the bacterial production of polyamines. (**D**) The microbial composition at genus level of the *in vitro* fermentation in Cellulose and Mucin groups. (**E**) Relative abundances of differential genera in Cellulose and Mucin groups. Statistics was performed with unpaired two-sided Student’s *t*-test. (**F**) The dominant genus in the *in vitro* fermentation by LEfSe analysis. (**G**) The dominant ASVs promoted in the *in vitro* fermentation by LEfSe analysis. The representative sequences of differential ASVs were further classified against the NCBI 16S rRNA database using blast software. (**H**) Heatmap between the polyamine concentrations and the relative abundance of top 20 genera in the *in vitro* fermentation. (*n* = 4); *P < 0.05, **P < 0.01, and ***P < 0.001.

Given that the effects of mucin on spermine accumulation were independent of host spermine synthesis, we investigated whether the gut microbiota was involved in enhanced spermine production. Similar to the spermine synthesis pathway in mammalian cells, partial intestinal microorganisms can synthesize spermine originally from arginine (23, 38, 39). Thus, the colonic bacterial 16S rRNA sequence was detected and analyzed. Compared with the FC and MUC diets, dietary fiber deprivation engendered a distinct microbial community (Fig. 2E; Fig. S4A), including a significantly reduced abundance of *unclassified_f_Atopobiaceae*, *Lactobacillus*, and *Bifidobacterium* (*P* < 0.01), as well as an increased *unclassified_f_Lachnospiraceae* abundance on the genus level (*P* < 0.05) (Fig. 2F; Fig. S4B). The MUC diet led to a significant enrichment of *unclassified_f_Atopobiaceae* and *Lactobacillus* (Fig. 3F; Fig. S4B). To further study the relationship between the composition of the gut microbiota and polyamine production, a correlation analysis was performed between the top 20 most abundant bacteria found in mucin-fed mice and the concentration of fecal polyamines. Interestingly, the correlation heatmap demonstrated that *Lactobacillus* had a positive relationship with spermidine and spermine (*P* < 0.05), as well as with the total polyamine levels (Fig. 2G), indicating that high-level *Lactobacillus* driven by mucin or fiber supplementation is a potential spermine-producing bacterium *in vivo*.

### Mucin drives high-level *Lactobacillus* species that is involved in the spermine accumulation during *in vitro* fermentation

To further confirm whether the enhancement of spermine synthesis by mucin supplementation occurred via reshaping of the microbial community, an *in vitro* batch fermentation system was established. Briefly, This batch system was used to explore the gut microbial dynamics and metabolites modulated by different fermentation substrates. As expected, mucin supplementation led to a dramatic increase in spermidine (*P* < 0.001) and spermine (*P* < 0.001) levels compared with those in the cellulose group (Fig. 3A). Next, the dose-dependent effect of mucin on spermine production was further investigated by replacing mucin from 2.5% to 10% in the cellulose. Mucin supplementation promoted spermine accumulation in a dose-dependent manner (*r* = 0.97, *P* < 0.01; Fig. 3B). Moreover, to verify the source of precursors for bacterial spermine synthesis, we compared the effects of full amino acid broths and arginine-deficient broths on spermine production *in vitro* fermentation. Under conditions of arginine deficiency (Arg-), the synthesis of bacterial spermine was significantly inhibited (*P* < 0.001), while the reduced spermine production was not reversed by mucin (Fig. 3C). Purified mucin, comprised of carbohydrates with different chemical properties, is structurally complex (40). To explore whether the components in mucin with slight structural differences could promote bacterial spermine metabolism, we employed four of the most abundant monosaccharides found in mucin and administered them in pure form to the microbial fermentation system. However, these four monosaccharides, including Gal, GalNAc, GlcNAc, and Fuc, and their mixtures all failed to affect the accumulation of spermidine and spermine (*P* < 0.001; Fig. S5). These results further revealed that mucin, rather than monosaccharide components, significantly increased the concentration of spermine. Collectively, we suggest that spermine production was achieved by enhanced microbial arginine metabolism by mucin.

To better understand the influence of mucin on the composition of the gut microbiota, we sought to determine the bacterial abundance after either cellulose or mucin administration during *in vitro* fermentation. Here, we observed moderate but significant changes in the microbiota composition between cellulose and mucin intervention (Fig. 3D). Specifically, mucin supplementation led to significant enrichment of *Lactobacillus*, *Escherichia-Shigella*, and *Bacillus* but decreased the abundance of *Prevotella* at the genus level (Fig. 3D through F). Given that the most abundant *Lactobacillus* found in mucin-fed mice was positively correlated with spermine production *in vivo*, we also analyzed the correlation between the levels of putrescine, spermidine, and spermine with the top 20 microorganisms at the genus level in *in vitro* fermentation. Similar results were observed in the Spearman correlation heatmap, where *Lactobacillus* was positively correlated with spermidine (*P* < 0.001) and spermine levels (*P* < 0.01; Fig. 3G). Overall, both *in vivo* and *in vitro* experiments revealed that *Lactobacillus* may contribute to spermine synthesis. We further employed LEfSe analysis at the ASV level in the *in vitro* fermentation experiment. Among all the used dietary fiber and the monosaccharides made up of mucin glycan, *L. mucosae* was identified as the most dominant *Lactobacillus* bacterium only in the presence of the mucin group (Fig. 3H; Fig. S6).

### *Limosilactobacillus mucosae*-derived spermine alleviates the colonic barrier defects only in mucin-fed mice

**Fig 4:**
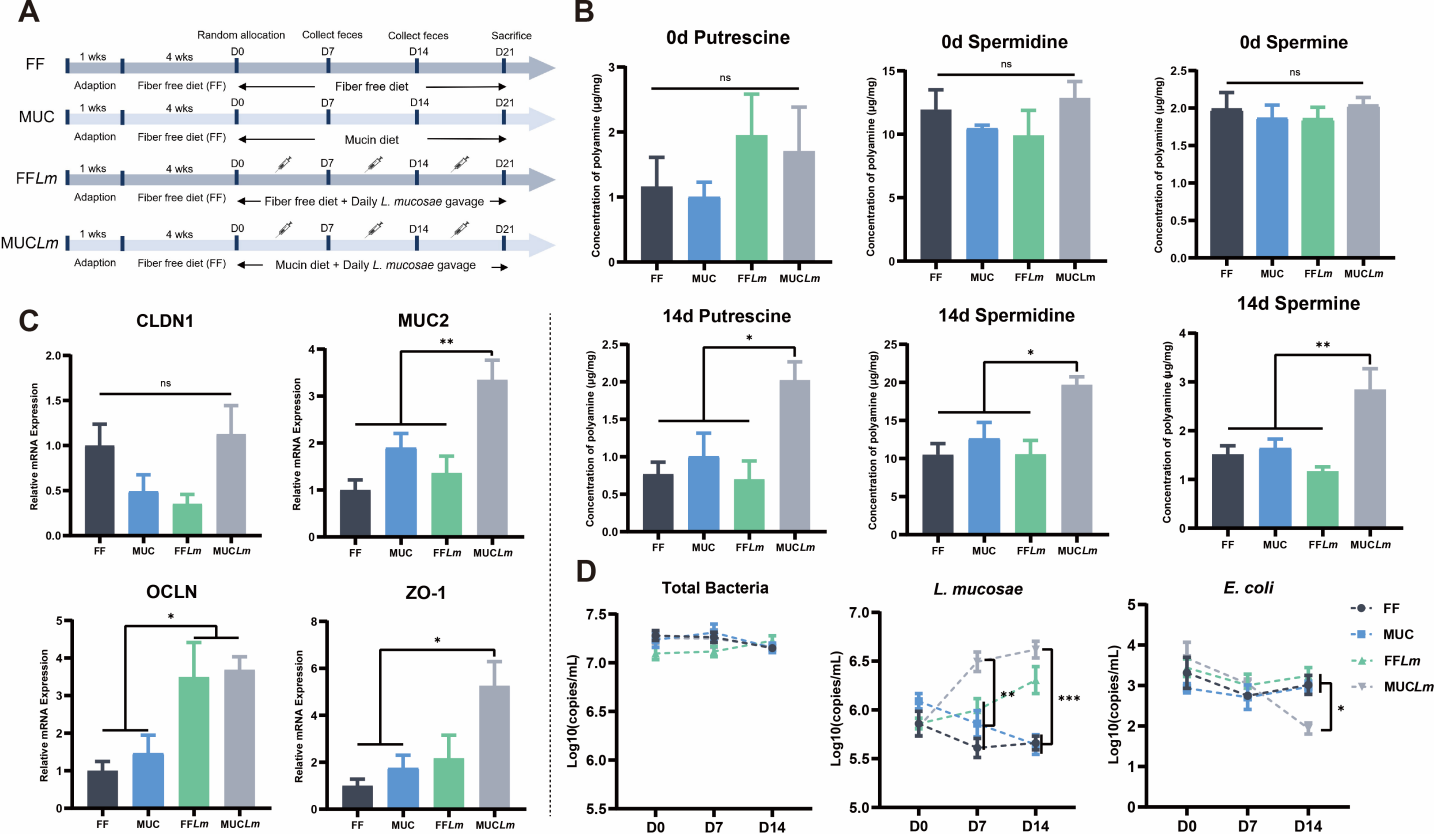
*Limosilactobacillus mucosae* alleviates the colonic barrier defects in mucin-fed mice. (**A**) Animal experiment set-up. (**B**) Concentrations of fecal polyamine at Day 0 and Day 14 (*n* = 6). (**C**) qPCR analysis of colonic barrier genes *Claudin-1*, *MUC2*, *Occludin*, and ZO*-1* from mice (*n* = 6). (**D**) Absolute copies of total bacteria, *L. mucosae*, and *E. coli* in the mouse feces at Day 0, Day 7, and Day 14 (*n* = 6). *P < 0.05, **P < 0.01, and ***P < 0.001.

To assess the potential of *L. mucosae* in alleviating intestinal homeostasis dysregulation due to dietary fiber deficiency, we transitioned mice from the FF diet to the MUC diet after 4 weeks feeding, while simultaneously administering daily *L. mucosae* gavage for 3 weeks. Prior to *L. mucosae* gavage, we first assessed baseline fecal polyamine levels and microbial compositions. Following *L. mucosae* gavage, mucin-fed mice exhibited significantly higher fecal spermine content compared with all other groups (*P* < 0.01; Fig. 4B). Additionally, the mRNA expression levels of intestinal barrier genes (MUC2, OCLN, and ZO-1) in colonic tissue were significantly elevated in the MUC*Lm* group compared with other groups (Fig. 4C). After 7 days, the abundance of *L. mucosae* decreased in both mucin-fed and fiber-free diet mice. Importantly, we found that under *L. mucosae* gavage condition, mucin-fed mice exhibited significantly higher *L. mucosae* colonization compared with fiber-free diet mice (*P* < 0.01; Fig. 4D), underscoring the critical role of mucin in enhancing *L. mucosae* colonization in the mouse colon. Furthermore, gavage of *L. mucosae* in mucin-fed mice significantly reduced the abundance of negative microorganisms, such as *E. coli* (*P* < 0.05), in the colon, indicating the significant impact of mucin and *L. mucosae* on the regulation of intestinal microecology. Collectively, these results emphasize that mucin can promote *L. mucosae*-derived spermine accumulation and highlight the potential of *L. mucosae* in mitigating intestinal dysregulation combined with mucin.

### *Limosilactobacillus mucosae* specializes in adhering on mucin without bacterial degradation

**Fig 5:**
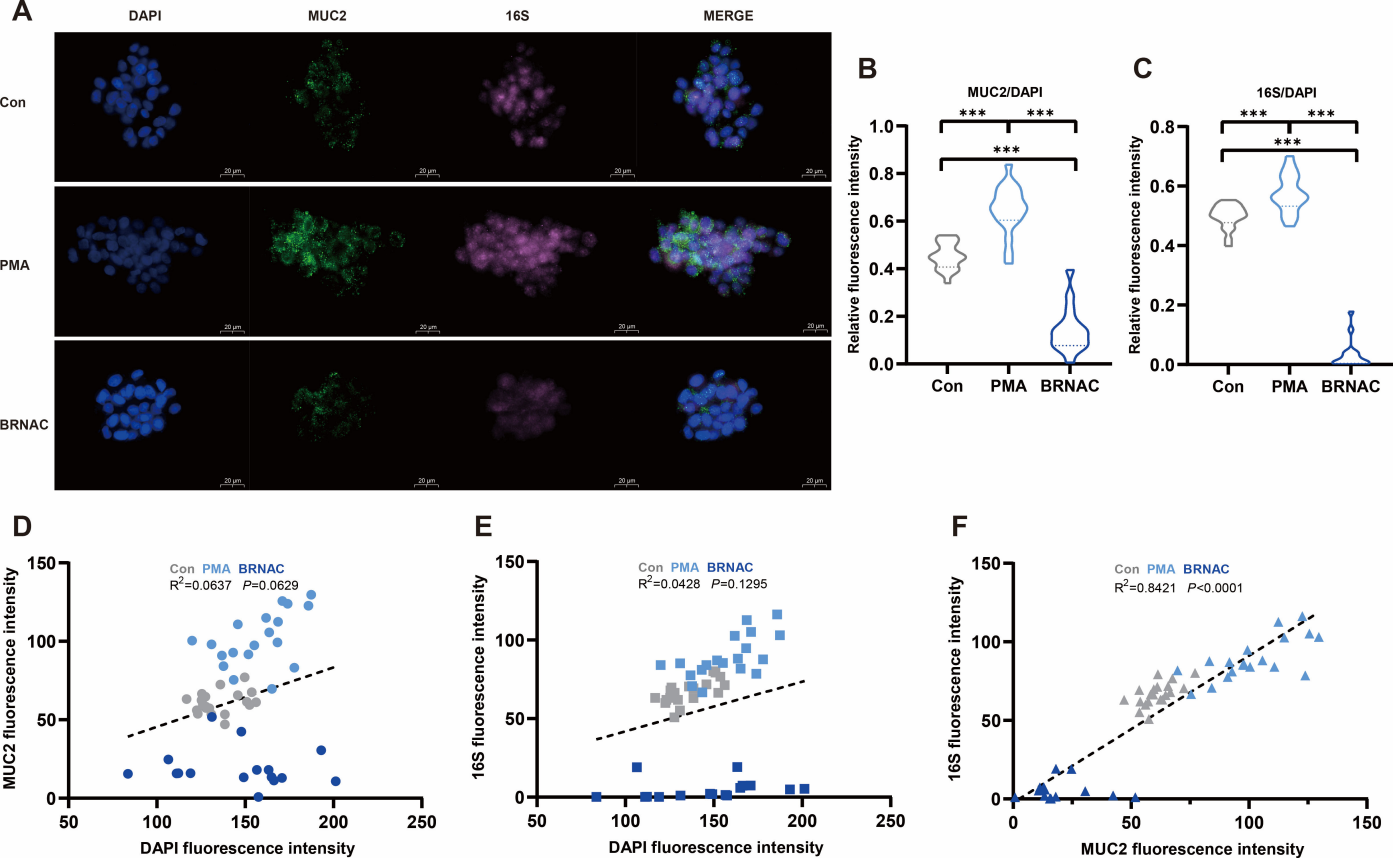
*Limosilactobacillus mucosae* colonizes on LS174T cells with dependence on mucin secretion. (**A**) Microscopy to take a fluorescent smear of cells secreting MUC2 and bacterial adhesion. (**B–C**) Statistics of the relative fluorescence intensity of labeled MUC2 protein and 16S rRNA gene; 15 fields of view were randomly selected for each smear to be counted. (**D–F**) Linear regression analysis of the fluorescence intensity of labeled MUC2 protein and 16S rRNA gene. *P < 0.05, **P < 0.01, and ***P < 0.001.

To investigate how mucin enriches *L. mucosae*, we induced mucin secretion by *in vitro* treatment with PMA (a mucin stimulator) and removed mucin from the cell surface by BRNAC (a mucin scavenger) in LS174T cells*—L. mucosae* co-cultured model. It was observed on the cell smears that the relative fluorescence intensity of MUC2 was significantly increased in PMA-treated cells, while decreased in the BRNAC group (*P* < 0.001; Fig. 5A and B). Consistently, similar results were obtained for the number of *L. mucosae* adhering to mucin tagged by 16S FISH after PMA or BRNAC treatment (*P* < 0.001; Fig. 5A and C). Next, we conducted a linear regression analysis between the relative fluorescence intensity of MUC2, 16S, and DAPI (Fig. 5D through F). Strikingly, only one significant linear relationship was detected between 16S and MUC2 (*P* < 0.001; Fig. 5F), indicating a significant positive linear correlation between mucin secretion and the number of *L. mucosae*.

**Fig 6:**
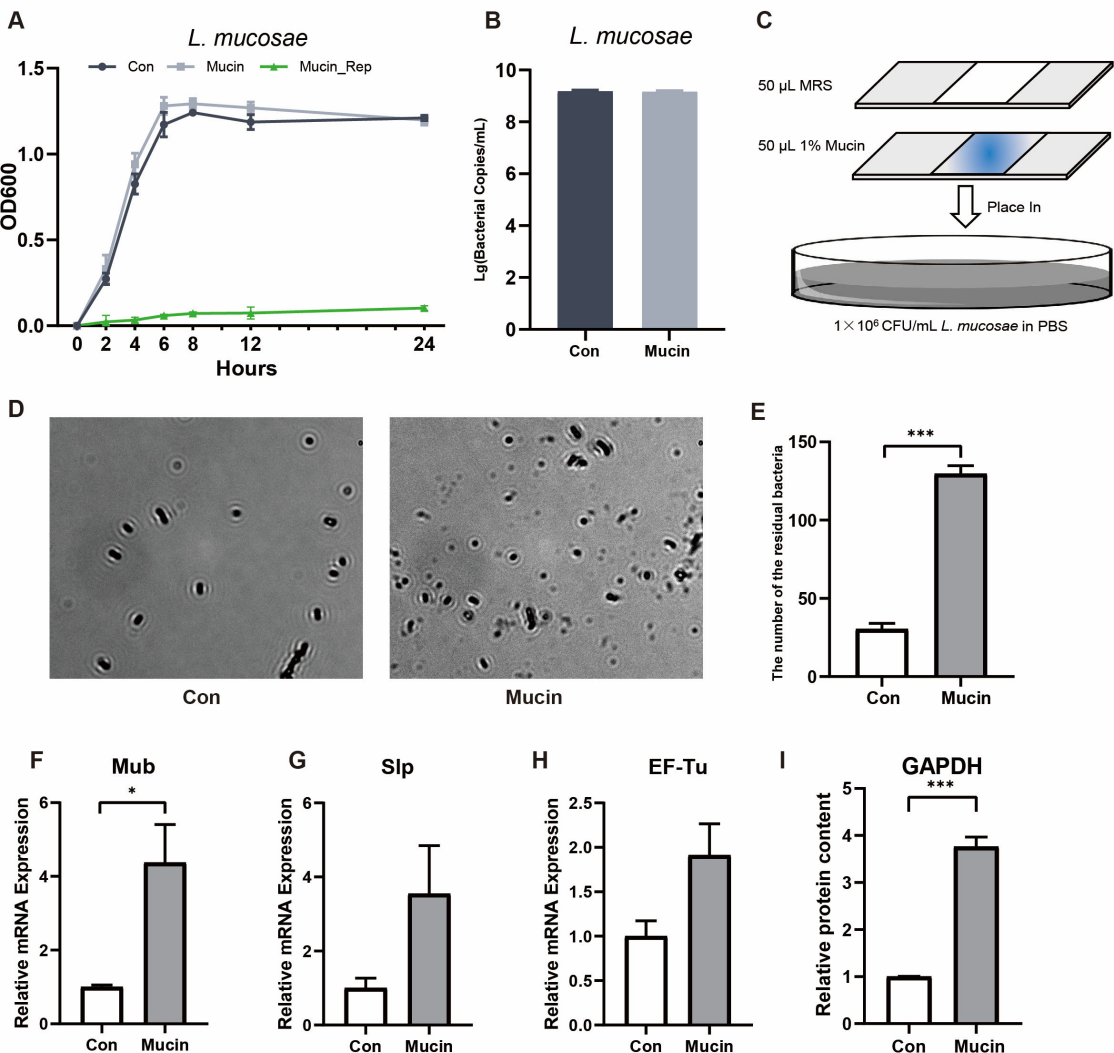
*Limosilactobacillus mucosae* adheres on mucin without bacterial degradation. (**A**) Quantitative measurement of *L. mucosae* in the MRS broth with or without mucin. (**B**) Growth curve of *L. mucosae* with or without mucin. (**C**) Schematic diagram of bacterial adhesion test. (**D**) Representative microscopy images of *L. mucosae* adhering to mucin-covered glass slides. (**E**) Quantification of the microscopy images (*n* = 3). Ten fields of vision were randomly selected under an oil microscope to calculate the number of bacteria adhered to the surface. Magnification = 1,000×. (**F–H**) qPCR analysis of the adhesive genes in *L. mucosae*. (**I**) Western blot analysis of bacterial surface protein GAPDH. *P < 0.05, **P < 0.01, and ***P < 0.001.

To further explore the potential interaction between mucin and microorganisms, *L. mucosae* was cultured in MRS broth containing mucin. However, 1% mucin supplementation failed to increase the growth rate or bacterial quantities (Fig. 6A and B). Notably, *L. mucosae* stops to proliferate when mucin was replaced with glucose in the MRS broth (Fig. 6A). Consistently, the bacteria were also unable to degrade mucin in 0, 8, and 24 h by sequential staining of glycoproteins that indicated that *L. mucosae* lacked mucin-degrading enzymes (Fig. S7). To verify the non-depleting effect of mucin on bacteria. We then measured the binding ability of mucin by counting the residual bacteria remaining on the smear (Fig. 6C) and found that the number of residual bacteria on the smear from mucin-containing broth was significantly higher than that in the control group. Moreover, a significant upregulation in the mRNA quantitation of bacterial surface adhesion genes (*Mub*, *Slp*, and *EF-Tu*) was also observed to be induced by mucin, as well as the quantitative analysis of the adhesion protein GAPDH in *L. mucosae*’s surface (Fig. 6F through I). These results suggest that mucin promotes the adhesion of *L. mucosae*.

### Mucin promotes spermine synthesis in *Limosilactobacillus mucosae* with a dependence on the enhanced arginine metabolism

**Fig 7:**
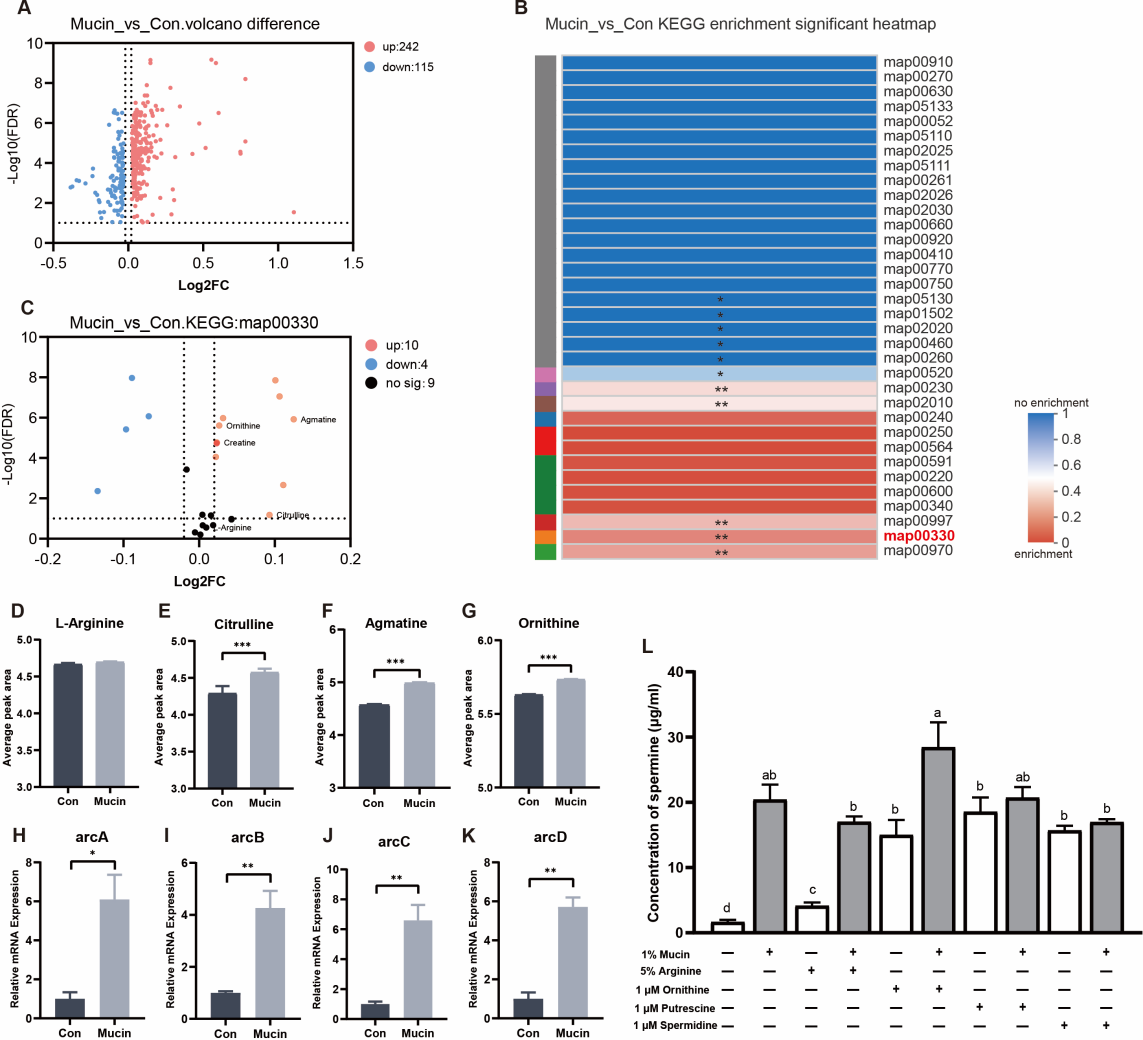
Mucin promotes spermine synthesis in *Limosilactobacillus mucosae* with a dependence on enhanced arginine metabolism. (**A**) Volcano plot of differential metabolites with or without mucin after *in vitro* culture of 8 h. (**B**) Heatmap of KEGG pathway enrichment by bacterial metabolites with or without mucin. The value is closer to 0, the and metabolite concentration in the metabolic set is enriched. If the metabolic set is not involved in this pathway, it is expressed as 1. (**C**) Volcano plot of differential metabolites belonging to pathway map00330 (arginine and proline metabolism) with or without mucin. The relative-betweenness centrality algorithm was used to obtain the pathway that was significantly enriched in the metabolite set, and the FDR (BH) method was used to correct the *P* value. (**D–G**) The concentration of arginine, citrulline, agmatine, and ornithine in control and mucin groups. (*n* = 4). (**H–K**) qPCR analysis of arginine metabolism gene cluster in *L. mucosae*. (**L**) The concentration of putrescine, spermidine, and spermine concentrations of *L. mucosae* culture in the MRS broth with the combination of mucin and metabolites in arginine metabolism. The letters at the top of the bar plot indicate statistically significant differences between the groups (*P* < 0.05). *arcA*, arginine deiminase; *arcB*, ornithine carbamoyltransferase; *arcC*, carbamate kinase; *arcD*, arginine/ornithine reverse transporter. Statistics was performed with one-way ANOVA, followed by Tukey’s multiple comparison test. *P < 0.05, **P < 0.01, and ***P < 0.001.

We previously verified that the source of increased spermine was derived from microbial metabolism, indicating the possibility that mucin could promote arginine metabolism in *L. mucosae*. To understand the specific role of mucin in arginine metabolism in *L. mucosae*, metabolome analysis was performed to identify differential metabolites driven by mucin supplementation. In total, 242 significantly upregulated metabolites and 115 downregulated metabolites were observed in the mucin group compared with the control group (Fig. 7A). The predicted KEGG functional enrichment analysis of the altered metabolites was associated with arginine and proline metabolism (map00330) (*P* < 0.01; Fig. 7B and C). Consistently, of the top 200 metabolites identified, metabolites belonging to the major subclusters were enriched in the mucin group (Fig. S8), illustrating enhanced metabolic activity within the strain modulated by mucin. There was no significant difference in arginine concentrations between the mucin group versus the control group illustrating that arginine in the medium is not deficient (Fig. 7D). Interestingly, mucin supplementation increased the concentration of metabolites related to arginine metabolism, such as citrulline, agmatine, and ornithine (*P* < 0.001; Fig. 7E through G). This result was verified by the transcriptional profiles of genes related to arginine metabolism in *L. mucosae*. The expressions of genes encoding arginine metabolic enzymes, including *arcA*, *arcB*, *arcC*, and *arcD* involved in the metabolism from arginine to ornithine, was significantly enriched in mucin-supplemented supernatant (Fig. 7H through K). Furthermore, to identify whether the spermine synthesis pathway was dependent on microbial arginine metabolites in *L. mucosae*, we cultured the bacteria in MRS medium supplemented with mucin and intermediate metabolites arginine, ornithine, putrescine, and spermidine. The results showed that the concentration of spermine was increased by administration of ornithine, putrescine, and spermidine alone, which was able to enhance the promoting effects of spermine accumulation caused by mucin, indicating that the role of mucin might be to promote microbial metabolism from arginine to ornithine (Fig. 7L). The above results suggest that spermine synthesis in *L. mucosae* is driven by mucin by promoting the enhanced activity of bacterial arginine metabolism.

### Increased spermine production of *Limosilactobacillus mucosae* driven by mucin improves the intestinal barrier function

**Fig 8:**
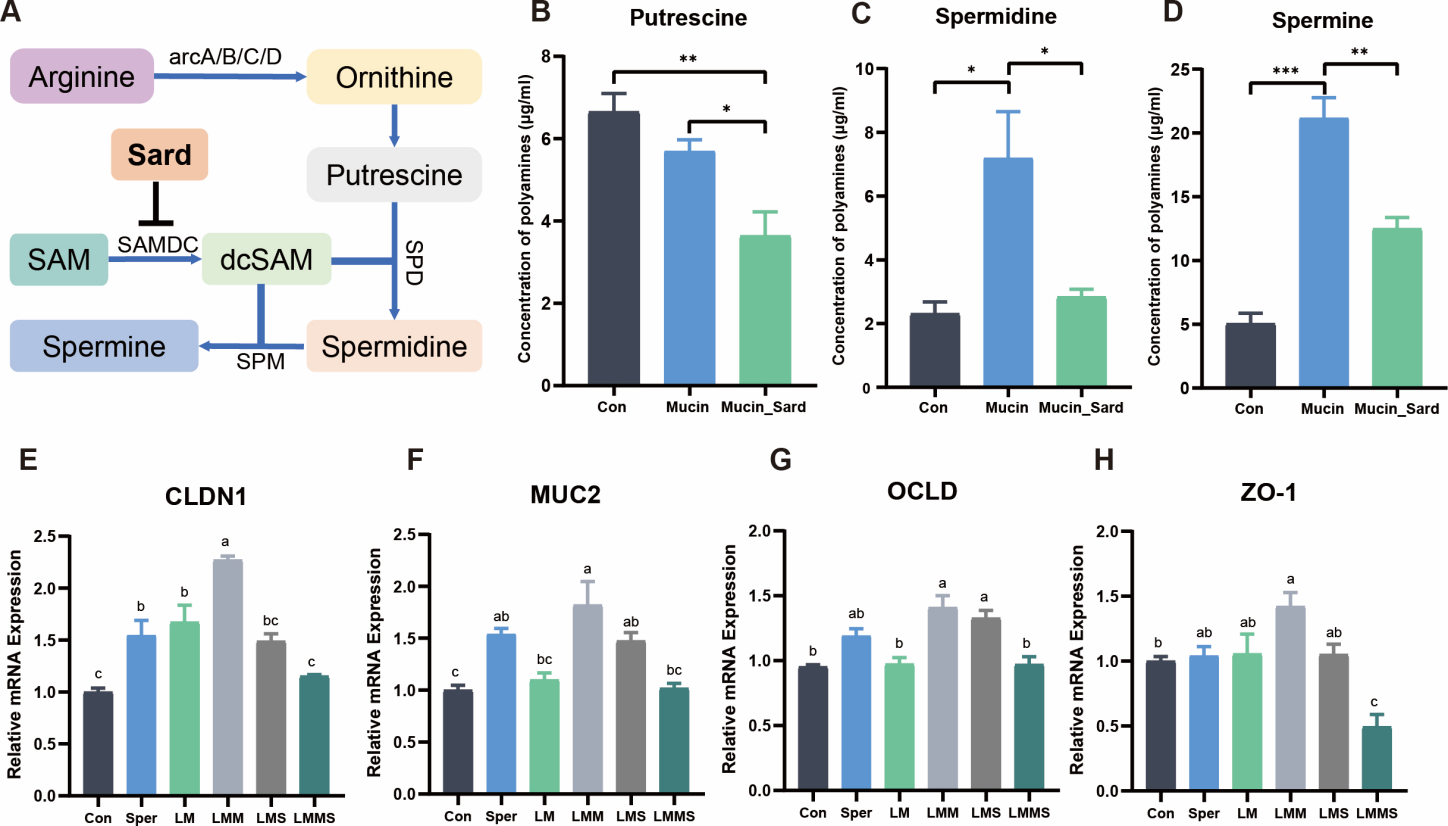
The increased spermine derived from *Limosilactobacillus mucosae* is verified to improve the barrier functions. (**A**) Schematic diagram of *L. mucosae* arginine metabolism. (**B–D**) The concentration of putrescine, spermidine, and spermine concentrations of *L. mucosae* culture in the MRS broth with or without mucin and sardomozide dihydrochloride. (**E–H**) qPCR analysis of tight-junction protein expression in LS174T cells. The letters at the top of the bar plot indicate statistically significant differences between the groups (*P* < 0.05). LM, normal culture supernatant; LMM, co-cultured supernatant with mucin; LMS, co-cultured supernatant with 8 µM spermine; LMMS, co-cultured supernatant with mucin and sardomozide dihydrochloride; Sper, 8 µM spermine.

As *Limosilactobacillus mucosae* was the key species in mucin-mediated spermine production, it was obvious to identify whether these bacteria were capable of producing spermine. The arginine metabolic pathway of *Lactobacillus* spp. to synthesize spermine was mapped according to previous research (Fig. 8A) (23, 41). Notably, mucin supplementation did not alter putrescine production in *L. mucosae* (Fig. 8B). More importantly, we found that mucin enhanced spermidine and spermine levels (Fig. 8C and D). To determine whether bacteria in response to mucin would drive spermine accumulation, sardomozide dihydrochloride (Sard), an inhibitor of polyamine synthesis, was added to MRS broth. Our results demonstrated that 10 µM Sard significantly reduced the concentration of spermine (*P* < 0.001) after mucin supplementation (Fig. 8D).

First, we determined the appropriate addition concentration of spermine in LS174T cells through 2 ~ 128 µM spermine addition (Fig. S9). The experimental results showed that when the spermine concentration was increased to 8 µM, the cell viability would not increase significantly. Therefore, in the subsequent study, 8 µM spermine was selected as the positive control concentration. To measure and assess the benefits of microbial-synthesized spermine on intestinal barrier function, either the normal bacterial supernatant (LM), 8 µM spermine (Sper), or the combination of bacterial supernatant and 8 µM spermine (LMS) was added to LS174T cells. As expected, the co-culture supernatant of *L. mucosae* and mucin (LMM) improved the mRNA expression of *CLDN1*, *MUC2*, and *OCLN* compared with the group in which spermine synthesis was inhibited (LMMS) and the LM group (Fig. 8E through H). Here, we found that the combination of spermine and the supernatant partially improved the cell barrier functions except the expression of *CLDN1*. Importantly, there were no significant differences in barrier function between Sper and LMS, indicating that the spermine was the certain key chemical that improved the intestinal barrier functions in the intestinal cells.

## DISCUSSION

The current study revealed that dietary fiber deprivation could lead to dysbiosis in colonic barrier protection. Consistent with previous work focused on the detrimental effects of microbial degradation on the host mucus layer (42, 43), mucin provision exogenously would reduce the consumption of the host mucus in the absence of dietary fiber (44). Hence, these findings support the idea that mucin-symbiotic capabilities are selected during host development and throughout the evolution of the host-microbe relationship to promote microbial stability (36). Accordingly, dietary administration of mucin could reverse intestinal health adversity, accompanied by re-stabilization of polyamine homeostasis in the host colon. It has been widely reported that polyamines are involved in various biological processes and therefore have important implications in promoting gut barrier function and regulating intestinal inflammation (19, 45–47). Previous studies have focused on the effects of dietary polyamines rather than microbial-derived polyamines on gut health in humans and animals (20, 38, 48). Fritsch et al. found that macrophages influence the growth and renewal of the colon epithelium by producing spermine, which enhances their proliferation and metabolism, especially during inflammation and stress (47). So, the microbial produced spermine should have similar effects as macrophage produced spermine on the colon epithelium, such as stimulating proliferation and barrier functions. In this study, we investigated whether host-derived or microbial-produced polyamines are critical for colonic barrier function. We provide the first evidence that the enhancement of spermine accumulation in mucin-fed mice is not achieved by regulating host spermine synthesis. Mucin-associated bacterial communities are also critical for promoting probiotic colonization resistance and determining disease pathology (49). Here, we observed that dietary fiber deprivation shifted the abundance of probiotics like *Lactobacillus* and *Bifidobacterium* toward the near extinction condition. One previous study in our lab also highlighted that a fiber-free diet can lead to the growth inhibition of beneficial bacteria and increased proliferation of pathogenic bacteria in a large animal model (33). Importantly, we figured out that the fiber-containing diet or the mucin-supplemented diet would simultaneously restore colonization of *Lactobacillus* and reinstate a new homeostatic balance in the colonic microbiome. Thus, this evidence elucidates the balance of intestinal mucin secretion and bacterial mucin degradation as important determinants of microbial distribution. By employing the correlation heatmap, *Lactobacillus* was selectively considered a potential bacterium for the synthesis of microbial spermine.

In an *in vitro* model of unraveling the microbial dynamic metabolic profiles and trophic roles of key microbes, mucin is indispensable in microbiome culture compared with other dietary carbon sources as it can regulate the microbe-microbe interaction and affect the metabolic profile of the microbiome (13). In this study, the *in vitro* batch fermentation model was used to monitor and gain insight into microbial metabolic responses to different source substrates. By employing *in vitro* experiments using diverse mediums, we demonstrated that direct supplementation with mucin, but not cellulose or other carbon sources, offered polyamine synthesis-promoting benefits, as evidenced by increased spermidine and spermine production. Notably, *Lactobacillus* was the dominant polyamine-synthesizing bacterium that could induce the production of multiple polyamines in the gastrointestinal tract (20). In the *in vitro* experiment, *Lactobacillus* was also positively correlated with the concentrations of spermine and spermidine, which was consistent with our results *in vivo*. It was also reconfirmed that *Lactobacillus* was involved in spermine production *in vitro*. Our current findings are in agreement with previous studies showing that the concentration of spermine and tyramine increased with *Lactobacillus helveticus* (41, 50). The pattern of amino acid utilization by different *Lactobacillus* species depends on its metabolic characteristics (51). Our data on *in vitro* fermentation supplementing mucin showed an enriched abundance of *Limosilactobacillus mucosae*, which partly revealed its preference for mucin. These findings are important for understanding bacterial-derived spermine as a critical signal, shedding light on gut health by establishing the proper function of the epithelial barrier. Nevertheless, it remains unknown how *L. mucosae* promotes spermine accumulation.

Co-culture of *Lactobacillus* and mucin has been reported to promote the colonization of *Lactobacillus* in a dynamic gut model and also increase their resistance to antibiotics (52–54). Another report confirmed that *Lactobacillus* could reduce the colonization of pathogenic bacteria in the intestinal mucosa through competitive adhesion (55, 56). Recently, whether mucin promoted the enrichment of *L. mucosae* or *L. mucosae* utilizing mucin as a functional substrate remains unclear. The adhesion of *L. mucosae* to host mucin has been shown to enhance gut health and relieve intestinal inflammation caused by *E. coli* infections (57, 58). The linear regression established between the concentrations of mucin and *L. mucosae* in two co-culture models confirmed a deeper connection in a non-consumption situation. Therefore, it is important to dissect and evaluate the relative contributions of mucin to the physiological metabolic changes of *L. mucosae* in spermine synthesis. In this study, we determined how mucin changed the various characteristics of *L. mucosae*. Mucin supplementation promoted amino acid activation during protein synthesis (map00970). Notably, it also significantly enriched the changes in metabolites in *L. mucosae*, which were related to arginine metabolism (map00330). Our results suggested that the production of spermine in *L. mucosae* after adding mucin and arginine is only significantly lower than that in the mucin + ornithine group, and there is no significant difference between the mucin group, ornithine/putrescine/spermidine, mucin + putrescine/spermidine. Therefore, we speculate that mucins should promote the transformation of arginine to ornithine, as the addition of ornithine and subsequent downstream metabolites have the effect of promoting spermine synthesis. These data indicate that the promoting effects of mucin on spermine accumulation depend on the microbial arginine metabolism in *L. mucosae.* Although we have shown that mucin, as an endogenous substance, changes the physiological properties of intestinal *Lactobacillus*, the underlying mechanisms triggered by mucin in bacteria should be elucidated in the future. Previous studies have reported that mucin O-glycans can influence the physiological behavior of microbes, including surface attachment, suppression of virulence genes, and regulation of quorum sensing signals (11, 12, 59). Given the complexity and diversity of mucin glycans and dynamic glycosylation changes based on different developmental stages, we posited that the presentation of complex mucin in the intestinal mucus contributes to a healthy mucosal environment, whereas degradation or modification of mucin may trigger *Lactobacillus* to transition arginine metabolism and further reduce its abundance (60, 61). Based on our current experimental results, it is evident that this study did not identify the specific genes regulated by mucin in the activation of spermine metabolism in *L. mucosae*. While our current experimental data demonstrate that *L. mucosae* metabolizes arginine to produce spermine, it is notable that no spermine synthase gene is annotated to any *Lactobacillus* species in the current public genome database. To address these gaps, in-depth whole-genome sequencing, assembly, and annotation of *L. mucosae* are necessary. Additionally, the knockout and targeted validation of potential spermine synthase genes in *L. mucosae* are required to elucidate the underlying mechanisms driving the protective effect of *L. mucosae* on the colonic barrier. These pending studies will lay the groundwork for prospective *in vitro* and pre-clinical investigations of intestinal microorganisms in the future.

**Fig 9:**
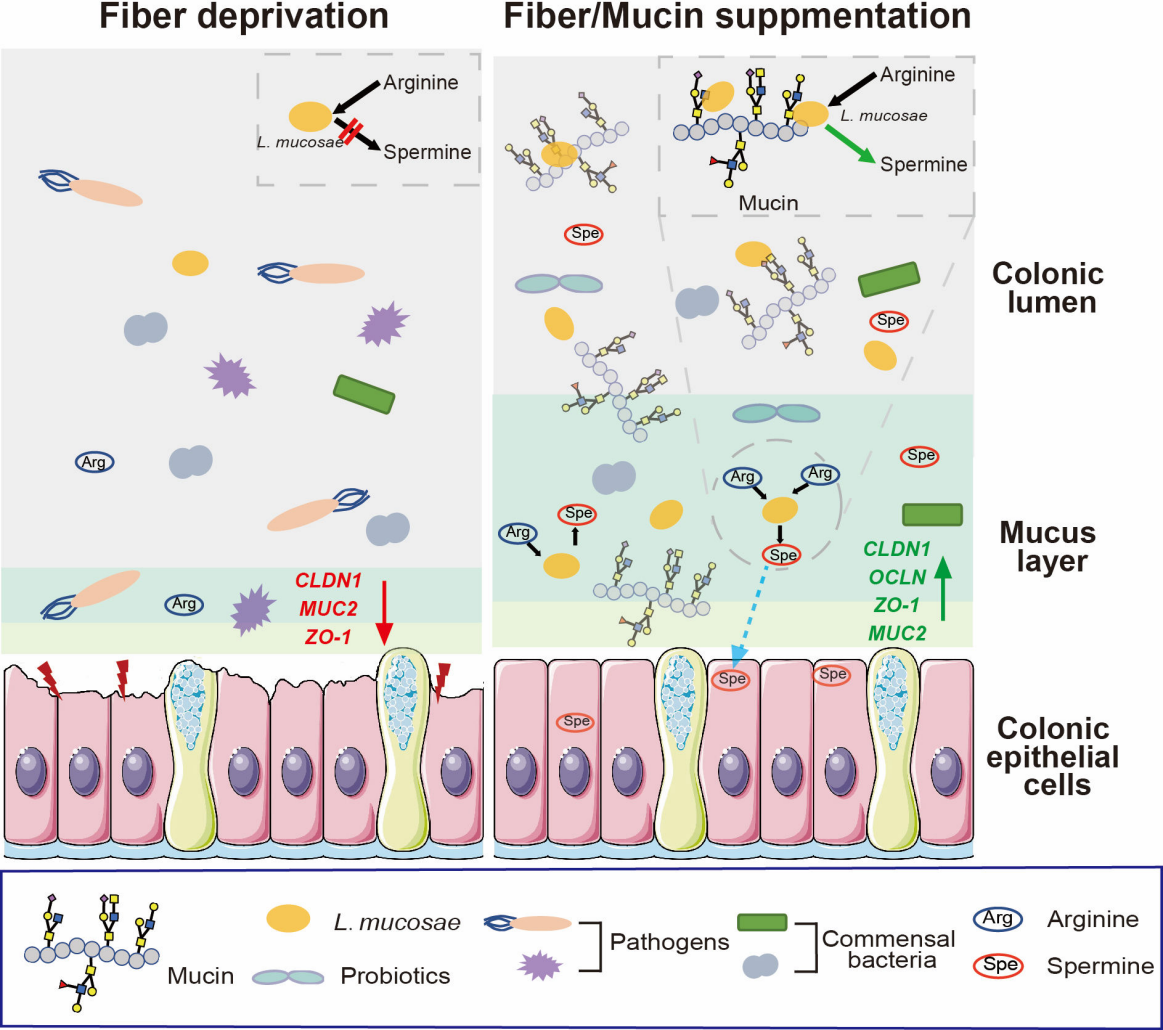
Schematic illustration of the spermine synthesis pattern in *Limosilactobacillus mucosae* modulated by mucin supplementation. Mucin rescues colonic barrier functions and probiotic extinction induced by dietary fiber deprivation, which leads to the accumulation of intestinal spermine level. *L. mucosae* enhances the activity of arginine metabolism to promote spermine synthesis depending on mucin-microbe crosstalk.

Overall, this study demonstrated that mucin rescued fiber deprivation-induced reduction of spermine accumulation in the colonic lumen and defects of the intestinal mucosal barrier. We indicated that mucin-induced changes in the metabolic characteristics of *Limosilactobacillus mucosae* in the enhanced arginine metabolism are an important prerequisite for the probiotic effects on relieving intestinal barrier disturbance in fiber-deprived mice and *in vitro* models (Fig. 9).

### Conclusion

In summary, we demonstrated that mucin mitigated dietary fiber deprivation-induced colonic barrier defects and decreased fecal spermine accumulation by reshaping the composition of the gut microbiota. Our study revealed that mucin plays a crucial role in promoting microbial spermine synthesis both *in vivo* and *in vitro*. Furthermore, *Limosilactobacillus mucosae* has been recognized as the potential bacterium that is positively associated with spermine accumulation. Consistent with expectations, spermine production by *L. mucosae* in mucin-fed mice contributed to the improvement of colonic barrier function. Specifically, *L. mucosae* promoted spermine synthesis with dependence on enhanced arginine metabolism through increased adhesion on mucin. These findings demonstrate the benefits of mucin supplementation to host gut health and its potential promoting effects on microbial arginine metabolism.

## Data Availability

The 16S rRNA sequences generated in this study are available in the NCBI Sequence Read Archive database (Accession Number: PRJNA847057).

## SUPPLEMENTAL MATERIAL

Supplemental figures msystems.00246-24-s0001.pdf Figures S1-S9.

Supplemental tables and legends msystems.00246-24-s0002.docx Tables S1 to S3 and legends to Fig. S1 to S9.

ASM does not own the copyrights to Supplemental Material that may be linked to, or accessed through, an article. The authors have granted ASM a non-exclusive, world-wide license to publish the Supplemental Material files. Please contact the corresponding author directly for reuse.
