# Supplementary figures and images for: Mucin alleviates colonic barrier dysfunction by promoting spermine accumulation through enhanced arginine metabolism in Limosilactobacillus mucosae
Source: mSystems. 2024 Apr 2;9(5):e00246-24. doi: 10.1128/msystems.00246-24 (PMC11097634; doi:10.1128/msystems.00246-24)

Figure S1

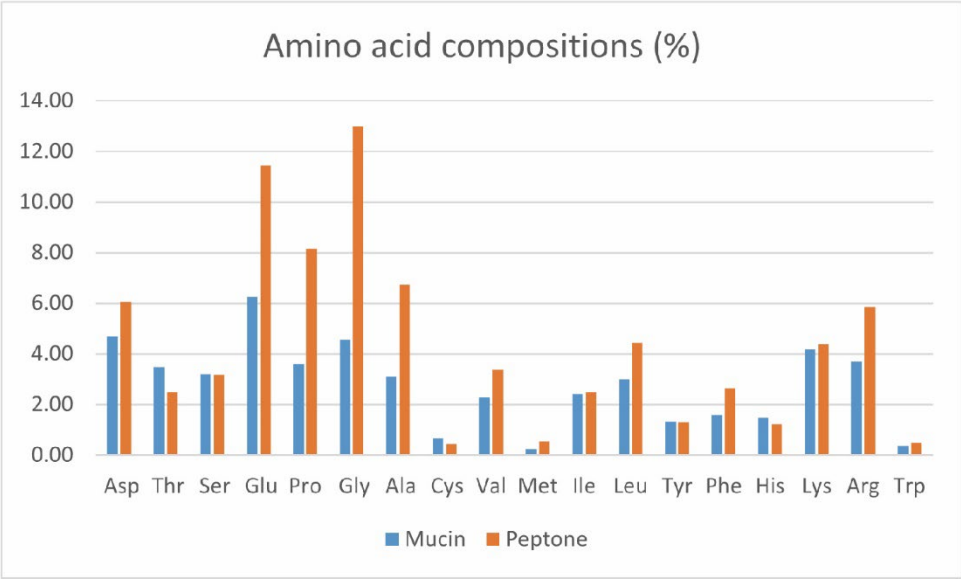

**Figure S2**

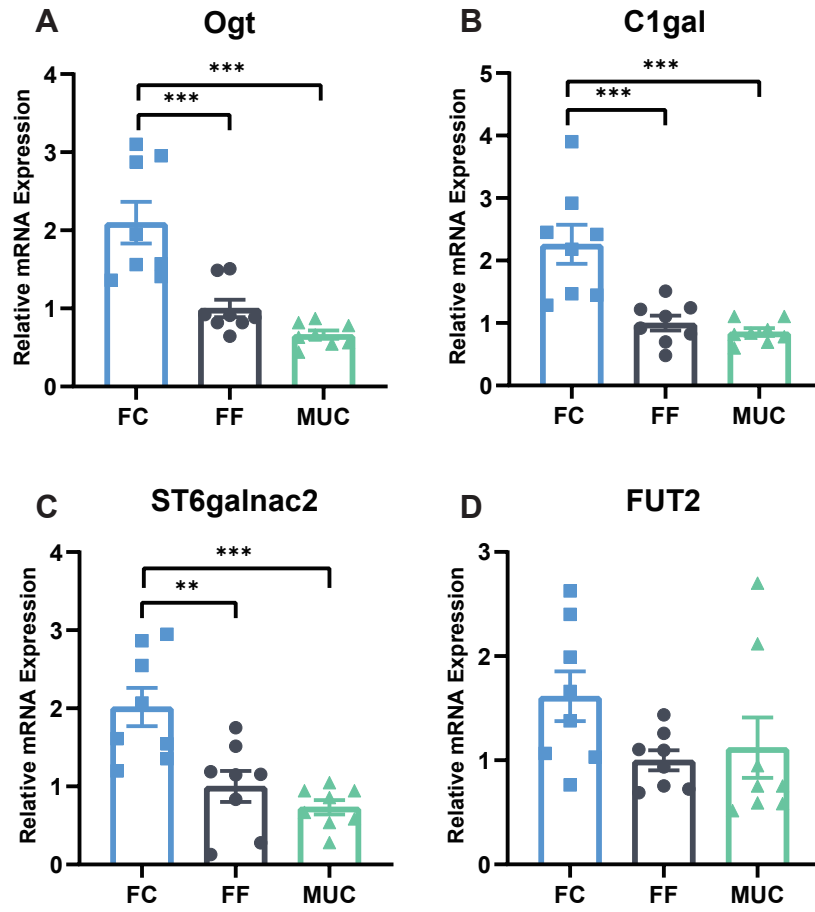

Figure S3

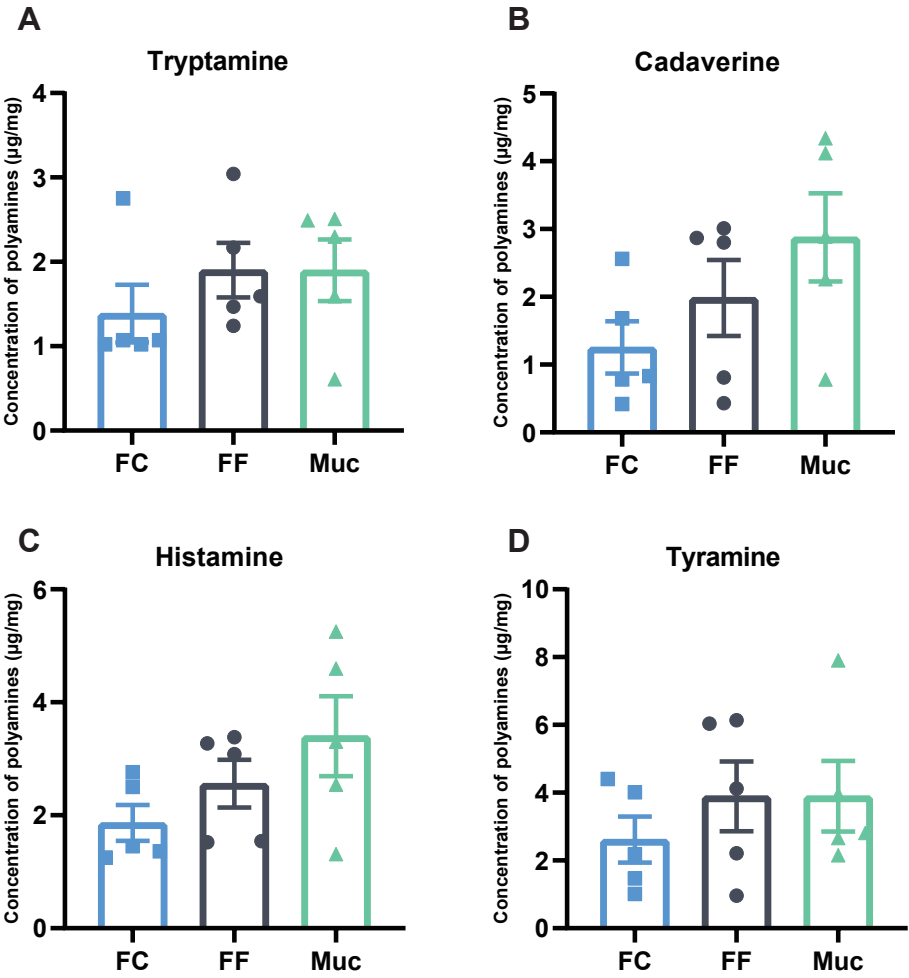

Figure S4

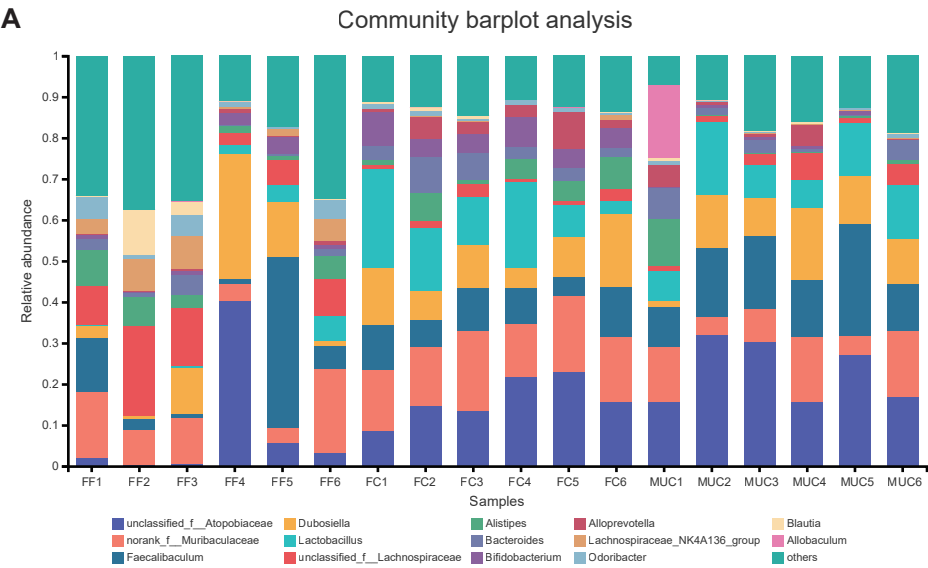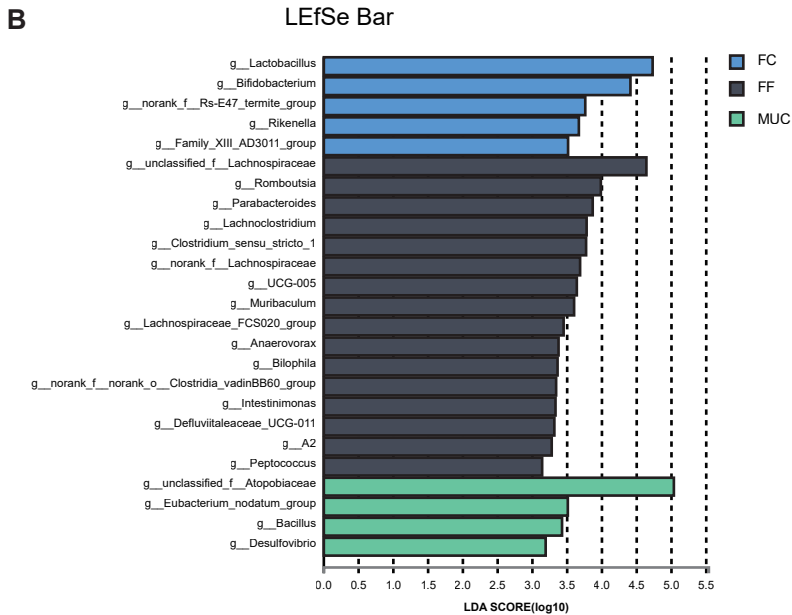

**Figure S5**

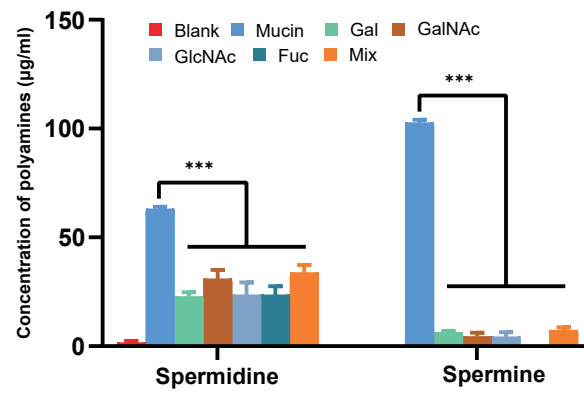

Figure S6

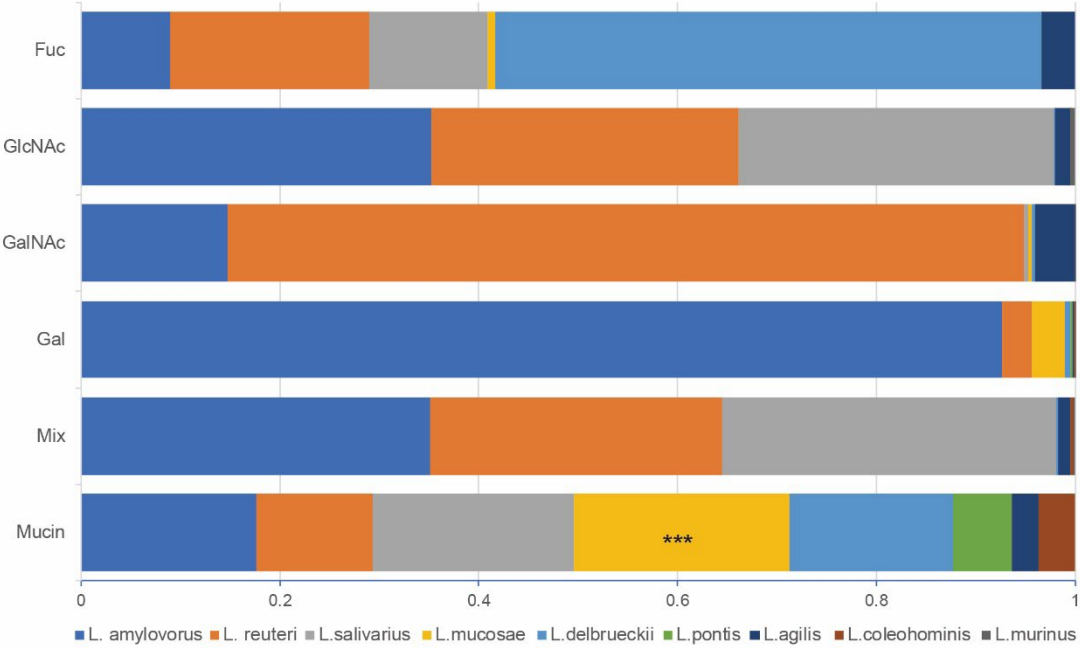

**Figure S7**

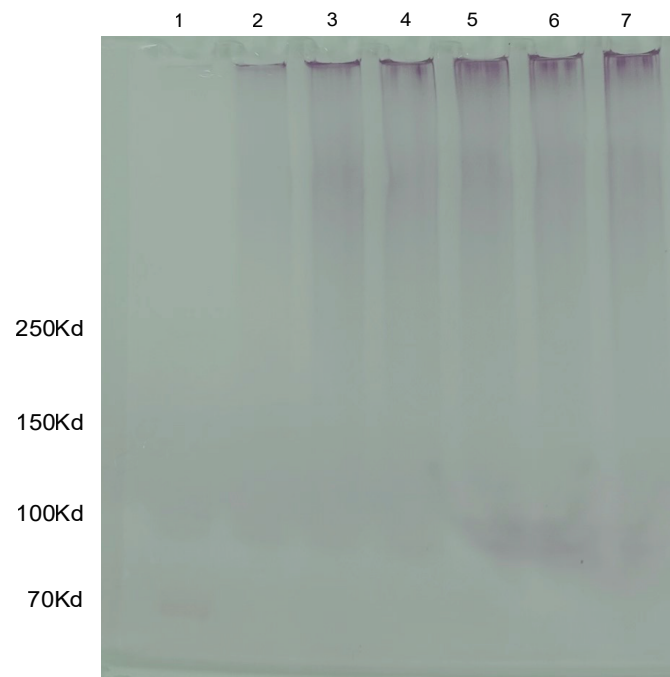

### Figure S8

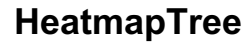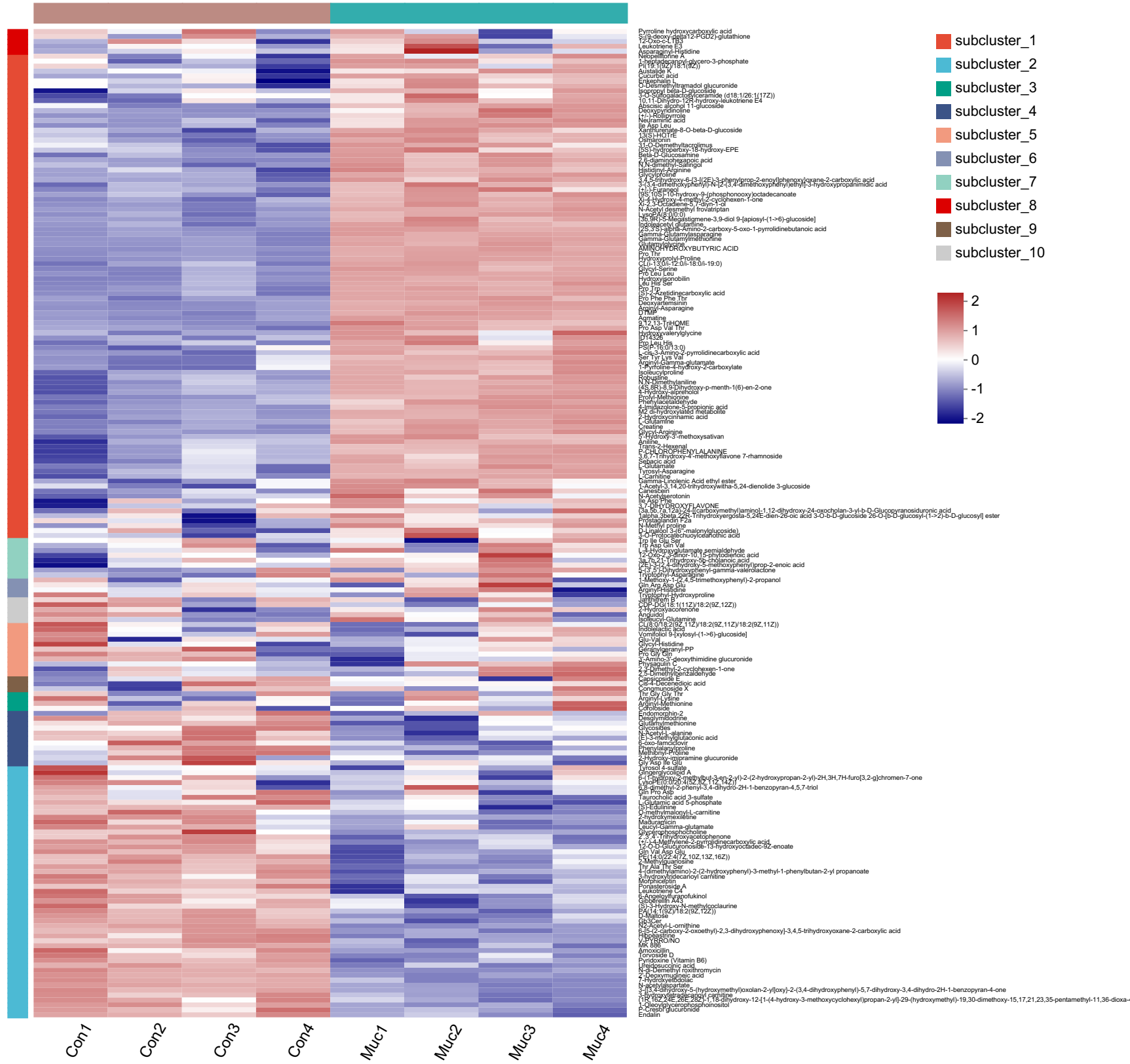

Figure S9

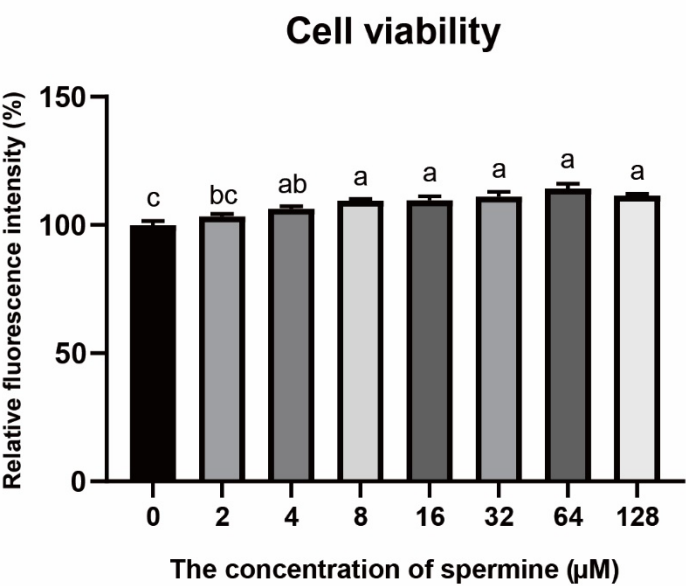

Supplement: Supplemental figures — Figures S1-S9. [file msystems.00246-24-s0001.pdf]
