# Supplementary material for: Mucin alleviates colonic barrier dysfunction by promoting spermine accumulation through enhanced arginine metabolism in Limosilactobacillus mucosae
Source: mSystems. 2024 Apr 2;9(5):e00246-24. doi: 10.1128/msystems.00246-24 (PMC11097634; doi:10.1128/msystems.00246-24)
Supplement: Supplemental tables and legends — Tables S1 to S3 and legends to Fig. S1 to S9. [file msystems.00246-24-s0002.docx]

| Fiber free diet |  | Fiber contained diet |  | Mucin diet |  |
| --- | --- | --- | --- | --- | --- |
| Ingredients | g/kg | Ingredients | g/kg | Ingredients | g/kg |
| Casein | 210 | Casein | 210 | Casein | 210 |
| L-cysstine | 3 | L-cysstine | 3 | L-cysstine | 3 |
| Maltodextrin | 50 | Maltodextrin | 50 | Maltodextrin | 50 |
| Surcose | 624.24 | Surcose | 574.24 | Surcose | 604.24 |
| Soybean Oil | 20 | Cellulose | 50 | Mucin Type II | 20 |
| Lard | 20 | Soybean Oil | 20 | Soybean Oil | 20 |
| Anhydrous Milkfat | 20 | Lard | 20 | Lard | 20 |
| AIN93G-MX(94046) | 35 | Anhydrous Milkfat | 20 | Anhydrous Milkfat | 20 |
| AIN93G-VX(94047) | 15 | AIN93G-MX(94046) | 35 | AIN93G-MX(94046) | 35 |
| Choline Bitartrate | 2.75 | AIN93G-VX(94047) | 15 | AIN93G-VX(94047) | 15 |
| TBHO | 0.01 | Choline Bitartrate | 2.75 | Choline Bitartrate | 2.75 |
|  |  | TBHO | 0.01 | TBHO | 0.01 |

**Table S1. Ingredient composition of the experimental diets**

**Table S2. HPLC gradient elution program for polyamine analysis**

| Time (min) | A % | B % |  |
| --- | --- | --- | --- |
| 0 | 40 | 60 |  |
| 5 | 40 | 60 |  |
| 12 | 25 | 75 |  |
| 20 | 5 | 95 |  |
| 21 | 40 | 60 |  |
| 25 | 40 | 60 |  |
| The flow rate:1.0 mL/min. A: ammonium acetate B: acetonitrile | | | |

**Table S3. Primer sequences used in RT-qPCR**

| Target genes | | Primers (5'-3') | |  |  | |  | |
| --- | --- | --- | --- | --- | --- | --- | --- | --- |
| Mouse |  |  |  |  |  | |  | |
| *MUC2* | Forward | CCTTAGCCAAGGGCTCGGAA | | | | |  | |
|  | Reverse | GGCCCGAGAGTAGACCTTGG | | | | |  | |
| *Claudin1* | Forward | GGGGACAACATCGTGACCG | | | | |  | |
|  | Reverse | AGGAGTCGAAGACTTTGCACT | | | | |  | |
| *ZO-1* | Forward | GCCGCTAAGAGCACAGCAA | | | | |  | |
|  | Reverse | TCCCCACTCTGAAAATGAGGA | | | | |  | |
| *Ogt* | Forward | ATTAGCAATGGACTGGCGACTACAC | | | | | | |
|  | Reverse | TGGGAACGGGTGGTTACAATAATGG | | | | | | |
| *Fut2* | Forward | AATTACAGGCGGTTCAAATGTC | | | | |  | |
|  | Reverse | AAACAATGTAGCATATTCGCCC | | | | |  | |
| *C1gal1* | Forward | TGGAATTACAACTATTATCCTCCCATA | | | | | | |
|  | Reverse | CAACATAGTGAAAAGAAACTGCGATA | | | | | | |
| *St6galnac2* | Forward | CCCACGAGCATTCTTTGACCCA | | | | | | |
|  | Reverse | TCAAACAGGCTGCGGAAGCGA | | | | |  | |
| *β-actin* | Forward | TGGAATCCTGTGGCATCCATGAAAC | | | | |  | |
|  | Reverse | TAAAACGCAGCTCAGTAACAGTCCG | | | | |  | |
| *L. mucosae* | |  |  |  |  | |  | |
| *arcA* | Forward | GTTACCAATCCAGCCCGCAGTAC | | | | |  | |
|  | Reverse | ATGACATCCCCGCCTTCCAGAG | | | | |  | |
| *arcB* | Forward | GCTGACTTTAACACCCGTGAAATGG | | | | |  | |
|  | Reverse | TCCAGACGCTTGCCTTCATTGC | | | | |  | |
| *arcC* | Forward | GTGCCAGCCGTTATCGACAAGG | | | | |  | |
|  | Reverse | ACATAGTCAACCGCCGTCAGAATG | | | | |  | |
| *arcD* | Forward | CTGAATCATCTCTTGCTCCGCTACC | | | | |  | |
|  | Reverse | GAAGCCAGCCAGTATGCCCAAC | | | | |  | |
| *EF-Tu* | Forward | GGTGCTATCTTAGTTGTTGC | | | | |  | |
|  | Reverse | CAACCAAGTCGATCAATTCT | | | | |  | |
| *Mub* | Forward | TGACCGGTGCCAGAAATCAG | | | | |  | |
|  | Reverse | CGTCAGTCGGATCATTGACCA | | | | |  | |
| *Slp* | Forward | GCACAACGCATACTACTACG | | | | |  | |
|  | Reverse | CTTGTCAACAGCCTTACCGT | | |  | |  | |
| *16S* | Forward | CACCGCTACACATGGAG | | |  | |  | |
|  | Reverse | AGCAGTAGGGAATCTTCCA | | | | |  | |
| LS174T cells | |  | | | |  |  |  |
| *Claudin-1* | Forward | TCTGGCTATTTTAGTTGCCACAG | | | | | |  |
|  | Reverse | AGAGAGCCTGACCAAATTCGT | | | | | |  |
| *Claudin-2* | Forward | CGGGACTTCTACTCACCACTG | | | | | |  |
|  | Reverse | GGATGATTCCAGCTATCAGGGA | | | | | |  |
| *Occludin* | Forward | ACAAGCGGTTTTATCCAGAGTC | | | | | |  |
|  | Reverse | GTCATCCACAGGCGAAGTTAAT | | | | | |  |
| *Mucin-2* | Forward | AACACAGTCCTGGTGGAAGG | | | | | |  |
|  | Reverse | CATTGTCAGGTCCCACACAG | | | | | |  |
| *ZO-1* | Forward | CAACATACAGTGACGCTTCACA | | | | | |  |
|  | Reverse | CACTATTGACGTTTCCCCACTC | | | |  |  |  |
| *VDR* | Forward | TCTCCAATCTGGATCTGAGTGAA | | | |  |  |  |
|  | Reverse | GGATGCTGTAACTGACCAGGT | | | |  |  |  |
| *GAPDH* | Forward | TGGTGAAGGTCGGAGTGAAC | | | |  |  |  |
|  | Reverse | GGAAGATGGTGATGCGATTTC | | | |  |  |  |

**Supplemental figure legends**

**Figure S1. Amino acid composition of bacterial peptone and mucin (%).**

**Figure S2. Mucin** **supplementation fails to influence mucin O-glycans synthesis in fiber-free mice.**

**A-D** qPCR analysis of the mucin synthesis genes (*Ogt*, *C1gal*, *ST6galnac2*, and *FUT2*) from the colons of mice (n = 8) fed with a fiber-contained diet (FC) a fiber-free diet (FF), and a fiber-free diet with mucin supplementation (MUC). Statistics was performed with one-way ANOVA, followed by Tukey’s multiple comparison test. *, *P* < 0.05; **, *P* < 0.01; ***, *P* < 0.001. *Ogt*: O-linked N-acetylglucosamine transferase. *C1gal*: core 1 synthase, glycoprotein-N-acetylgalactosamine 3-beta-galactosyltransferase 1. *ST6galnac2*: ST6 N-acetylgalactosaminide alpha-2,6-sialyltransferase 2. *FUT2*: fucosyltransferase 2.

**Figure S3. Mucin supplementation had no effects on fecal polyamine contents except for the polyamines produced by arginine metabolism.**

**A**-**D** The concentrations of fecal tryptamine, cadaverine, histamine and tyramine in the mice (n = 5). Statistics was performed with one-way ANOVA, followed by Tukey’s multiple comparison test.

**Figure S4. Mucin supplementation exhibits distinct microbial community compared with fiber-free mice.**

**A** Microbial composition on genus level of the mice feed with a fiber-contained diet (FC) a fiber-free diet (FF), and a fiber-free diet with mucin supplementation (MUC). **B** The dominant genus promoted by three dietary treatments by LEfSe analysis.

**Figure S5. The monosaccharides of mucin O-glycans are not responding to the production of microbial polyamines in *in vitro* fermentation.**

The concentrations of spermidine and spermine in the broth where different monosaccharides including Gal, GalNAc, GlcNAc and Fuc in mucin as the only carbon sources. Mix: The mix group was in a ratio of the five monosaccharides (Gal: GalNAc: GlcNAc: Fuc: Neu5Ac in 35: 25: 25: 10: 5). (n = 4), *, *P* < 0.05; **, *P* < 0.01; ***, *P* < 0.001. Gal: Galactose, Fuc: Fucose, GalNAc: N-Acetyl-D-galactosamine, GlcNAc: N-Acetyl-D-glucosamine, Neu5Ac: N-Acetylneuraminic acid.

**Figure S6. Mucin sugars and mixture failed to enrich *L. mucosae* among *Lactobacillus* species.**

The compositions of *Lactobacillus* species classified at the ASV level in mucin sugar mediums and the mixture medium in *in vitro* fermentation (n = 4).

**Figure S7. Mucin degradation enzymes are not expressed in *L. mucosae*.**

Mucin was resolved by SDS-PAGE and visualized using a specific glycoprotein staining. In all lanes, the protein precipitated in 10 ml of culture or solution, corresponding to approximately 40 µg of protein, and was further visualized using glycoprotein staining. Lane 1: Protein Ladder; Lane 2-3: 0 h culture supernatant; Lane 4-5: 8 h culture supernatant; Lane 6-7: 24 h culture supernatant.

**Figure S8.** **Mucin supplementation enriches more microbial metabolites in *L. mucosae*.**

Untargeted metabolome profile of microbial metabolites in *L. mucosae* with mucin supplementation cultured in 8 hours (n = 4).

**Figure S9. Effects of different layer concentrations of spermine on LS174T cells’ viability (n = 4).**
